# Optimization of the r^2^SCAN-3c Composite Electronic-Structure Method for Use with Slater-Type Orbital Basis Sets

**DOI:** 10.1021/acs.jpca.2c02951

**Published:** 2022-06-02

**Authors:** Thomas Gasevic, Julius B. Stückrath, Stefan Grimme, Markus Bursch

**Affiliations:** † Mulliken Center for Theoretical Chemistry, Universität Bonn, Beringstr. 4, D-53115 Bonn, Germany; ‡ Max-Planck-Institut für Kohlenforschung, Kaiser-Wilhelm-Platz 1, D-45470 Mülheim an der Ruhr, Germany

## Abstract

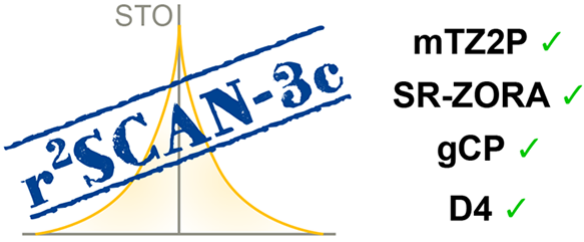

The “Swiss army knife” composite density functional electronic-structure method r^2^SCAN-3c (*J. Chem. Phys.***2021**, *154*, 064103) is extended and optimized for the use with Slater-type orbital basis sets. The meta generalized-gradient approximation (meta-GGA) functional r^2^SCAN by Furness et al. is combined with a tailor-made polarized triple-ζ Slater-type atomic orbital (STO) basis set (mTZ2P), the semiclassical London dispersion correction (D4), and a geometrical counterpoise (gCP) correction. Relativistic effects are treated explicitly with the scalar-relativistic zeroth-order regular approximation (SR-ZORA). The performance of the new implementation is assessed on eight geometry and 74 energy benchmark sets, including the extensive GMTKN55 database as well as recent sets such as ROST61 and IONPI19. In geometry optimizations, the STO-based r^2^SCAN-3c is either on par with or more accurate than the hybrid density functional approximation M06-2X-D3(0)/TZP. In energy calculations, the overall accuracy is similar to the original implementation of r^2^SCAN-3c with Gaussian-type atomic orbitals (GTO), but basic properties, intermolecular noncovalent interactions, and barrier heights are better described with the STO approach, resulting in a lower weighted mean absolute deviation (WTMAD-2(STO) = 7.15 vs 7.50 kcal mol^–1^ with the original method) for the GMTKN55 database. The STO-optimized r^2^SCAN-3c outperforms many conventional hybrid/QZ approaches in most common applications at a fraction of their cost. The reliable, robust, and accurate r^2^SCAN-3c implementation with STOs is a promising alternative to the original implementation with GTOs and can be generally used for a broad field of quantum chemical problems.

## Introduction

In recent years, Kohn–Sham density functional theory (DFT)^1^ has become one of the most popular methods in quantum chemistry, mainly due to its outstanding accuracy to computational cost ratio.^2^ It can be employed for a large number of problems, including molecular structures and various chemical properties, as well as reactions that facilitate research and commercial projects.^3−6^ Despite its high efficiency, the limits of conventional DFT are quickly reached for calculations of large systems that contain more than 300 atoms. The emerging need for fast yet accurate low-cost methods paves the way for composite schemes. These typically include small optimized basis sets to reduce the computational cost and compensate for the resulting errors with tailored corrections. A prominent class of such composite schemes is the “3c” method family. The first 3c method was the Hartree–Fock theory-based HF-3c^7^ method that contains three name-giving corrections to improve its accuracy. The same concept was later applied to DFT from which the PBEh-3c/HSE-3c^8−10^ hybrid and B97-3c^11^ GGA functionals resulted.

The latest addition to the “3c” family is r^2^SCAN-3c,^12^ which utilizes a well-balanced triple-ζ Gaussian-type atomic orbital (GTO) basis set, the D4 London dispersion correction,^13,14^ and a geometrical counterpoise (gCP)^15^ correction for remaining inter- and intramolecular basis set superposition errors (BSSE). The underlying meta-generalized-gradient approximation (meta-GGA)-type density functional r^2^SCAN^16,17^ is the regularized and restored form of the strongly constrained and appropriately normed (SCAN)^18^ functional. r^2^SCAN yields improved accuracy and a much reduced sensitivity to the employed numerical integration grid. Overall, the original GTO-based r^2^SCAN-3c was shown to yield excellent results for the calculation of thermochemical properties as well as conformational energies for systems with main-group elements and transition metals, partly reaching the accuracy of hybrid functionals, applying basis sets of quadruple-ζ (QZ) quality.^12^

Up to this point, the “3c” composite schemes were limited to GTO basis sets, while an assessment with Slater functions is missing. They satisfy Kato’s cusp condition^19^ at the nucleus and possess a correct long-range behavior. In this work, we present an optimized Slater-type atomic orbital (STO) variant of the composite r^2^SCAN-3c DFT method with a customized all-electron STO basis set which was implemented in the Amsterdam Density Functional (ADF) program^20,21^ of the Amsterdam Modeling Suite (AMS).^22^ The performance of r^2^SCAN-3c is compared for both implementations (GTO vs STO) and extensively assessed on a comprehensive database consisting of 621 data points for geometrical quantities and 4405 data points for energies. This data collection includes extensive benchmark sets such as the GMTKN55^23^ database as well as additional benchmark sets for noncovalent interactions (e.g., IONPI19^24^), conformational energies, and organometallic reactions (e.g., MOR41^25^ and ROST61^26^). Comparisons between GTOs and STOs have already been made in a different context,^27^ but this study is probably one of the most extensive ones considering the variety, chemical relevance, and amount of evaluated data points.

## Theoretical Methods

**Figure 1:**
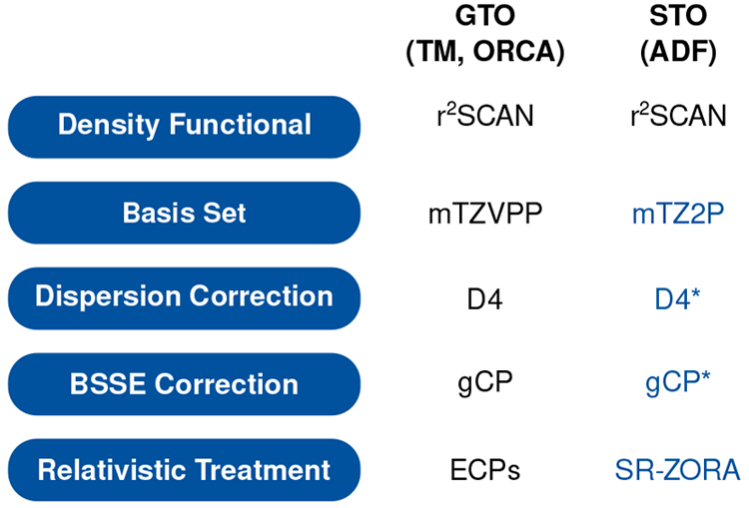
Components of r^2^SCAN-3c applying Gaussian-type atomic orbitals (GTO) and Slater-type atomic orbitals (STO). Changes in the STO approach are marked in blue. The D4 and gCP corrections were adjusted for STOs as indicated by an asterisk.

The composite electronic-structure method r^2^SCAN-3c consists of five different components, some of which are interdependent. An overview is shown in Figure 1, and modifications of each component will be discussed in the following. The main building block is the underlying density functional approximation (DFA) r^2^SCAN. In comparison to its predecessors SCAN and rSCAN,^28^ it is more accurate and less sensitive to the numerical integration grid. Consequently, much finer integration grids compared to those used in other conventional DFT methods are not required anymore, which leads to faster and more robust computations. In the STO approach, the basic DFA remains unchanged and is implemented via the Libxc library.^29^

### Basis Set Modification

The new modified all-electron triple-ζ basis set mTZ2P includes a combination of the default STO atomic orbital basis sets DZP, TZP, and TZ2P in ADF, which are contracted for the zeroth-order regular approximation (ZORA).^30−32^ It is constructed in analogy to the original GTO basis set mTZVPP to which it is compared in Table 1. The contraction schemes do not match for every element due to the different composition of the STO basis set. For example, one d- and one f-polarization function each are used for oxygen instead of two d-functions. In general, the STO basis sets include more basis functions for heavy elements compared to the original GTO basis set as the latter per default applies small-core effective core potentials (ECP) to represent the core electrons. Alterations to the underlying DZP and TZP basis sets by removing or exchanging basis functions (e.g., replacing f- by d-polarization functions) were not successful, typically increasing the obtained errors.

**Table 1:** Comparison of the New mTZ2P STO Basis Set with the Original mTZVPP GTO Set^a^

	contraction		underlying
element	mTZVPP	mTZ2P	STO basis
H	[2s1p]	[2s1p]	DZP^b^
He	[2s1p]	[2s1p]	DZP
N	[5s3p2d]	[5s3p2d]	TZ2P
O	[5s3p2d]	[5s3p1d1f]	TZ2P^c^
F	[5s3p2d]	[5s3p2d]	TZ2P
Ne	[5s3p2d]	[5s3p1d1f]	TZ2P
Si–S	[5s4p2d]	[7s5p1d1f]	TZ2P
Cl	[5s4p2d]	[7s5p1d1f]	TZ2P
Ar	[5s4p2d]	[7s5p1d1f]	TZ2P
Kr	[6s5p4d]	[8s7p4d1f]	TZ2P

a Elements that are not listed are described by the standard TZP basis in the STO set.

b 2p exponent is changed from 1.25 to 1.70.

c 3d exponent is changed from 2.00 to 2.15.

**Figure 2:**
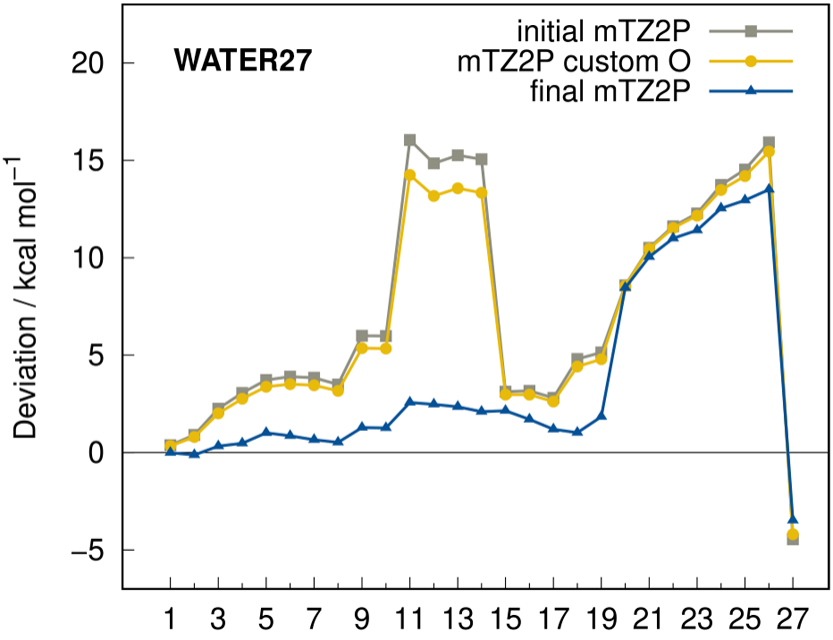
Deviations calculated with r^2^SCAN-3c(STO) for the WATER27 interaction energy benchmark set applying the original exponents for oxygen and hydrogen (initial mTZ2P), customized exponents for oxygen (mTZ2P custom O), and customized exponents for oxygen and hydrogen (final mTZ2P).

Nevertheless, it was found that the respective 3d- and 2p-polarization functions of oxygen and hydrogen were initially too diffuse (too small exponents) when they are used in combination with the D4 and gCP corrections. The exponents were manually optimized using the WATER27^33^ (water clusters), S22,^34^ S66^35,36^ (noncovalent interactions of small molecules), and HB300SPX^37^ (hydrogen bonds) benchmark sets. First, the 3d exponent of oxygen was changed from 2.00 to 2.15 and subsequently the 2p exponent of hydrogen from 1.25 to 1.70. The effect on the WATER27 set is illustrated in Figure 2. Here, the optimization of the 3d exponent of oxygen already improves the results noticeably but the influence of tuning the 2p exponent of hydrogen is more substantial, drastically decreasing the tentative underestimation of the intermolecular water–water interactions.

### London Dispersion Correction (D4)

**Figure 3:**
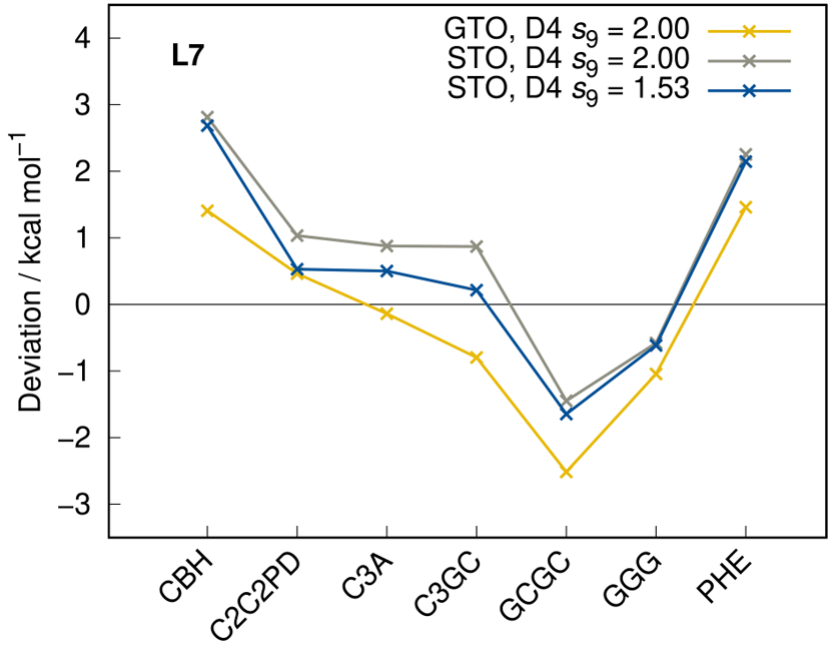
Deviations from reference values calculated with r^2^SCAN-3c for the L7 benchmark set applying different *s*_9_ D4 parameters.

Since semilocal density functional approximations do not account for long-range electron correlation effects, they lack the description of London dispersion interactions.^38,39^ In r^2^SCAN-3c, they are included by the atomic-charge dependent London dispersion correction D4, which is calculated according to
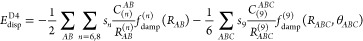
1where *A*, *B*, and *C* denote atoms, *s*_*n*_ is the scaling parameter, *C*_(*n*)_ is the dispersion coefficient, *R*_*AB*_ is the interatomic distance, *R*_*ABC*_ is the geometrically averaged distances, θ_*ABC*_ is the angle in atomic triangles, and *f*_damp_^(*n*)^ is the Becke–Johnson damping function *f*_BJ_^(*n*)^:
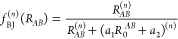
2with the functional specific parameters *a*_1_ and *a*_2_. As described in Basis Set Modification, the STO basis set mTZ2P is generally more diffuse than the GTO basis set mTZVPP and has a different long-range behavior due to the shape of the Slater-type functions. Accordingly, the manifestation of basis set superposition error (BSSE) is different for both basis sets which has a direct influence on the D4 and gCP corrections that have to be adjusted accordingly. The comparison between the GTO and STO variants of r^2^SCAN-3c in Figure 3 shows that, by applying the same D4 and gCP corrections (original parameters of the GTO variant), the interaction energies differ by up to 1.66 kcal mol^–1^ in the L7 benchmark set for noncovalent interactions of large complexes.^40,41^ Here, a slight mismatch of the attractive D4 correction and the repulsive gCP is observed for the STO basis set. To partly correct this issue, the *s*_9_ scaling parameter of the three-body dispersion has been set to 1.53 (vs 2.00 in the GTO approach), as it yields the lowest mean absolute deviation for the S30L benchmark set^42^ (association energies of large NCI complexes). The remaining parameters *s*_6_, *s*_8_, *a*_1_, and *a*_2_, as well as the parameters in the charge-scaling functions β and γ (see eq 2 of ref (14)) are kept unchanged. An overview of the utilized D4 parameters is listed in the Supporting Information. The effect of adjusting the *s*_9_ parameter for the L7 set is depicted in Figure 3. In general, the BSSE at the TZ basis set level can be partly absorbed in the D4 parametrization.^43^

### Geometrical Counterpoise Correction (gCP)

**Figure 4:**
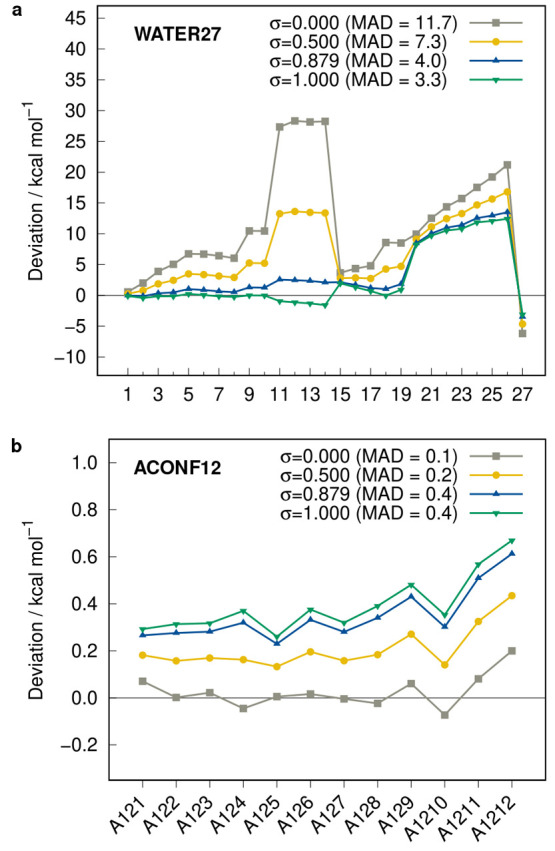
Deviations calculated with r^2^SCAN-3c(STO) for the WATER27 (a) and ACONF12 (b) benchmark sets applying different settings for the global scaling σ of the gCP correction. MADs are given in kcal mol^–1^.

Calculations applying finite basis sets are contaminated by inter- as well as intramolecular BSSE. These errors can be corrected with a geometrical counterpoise scheme according to
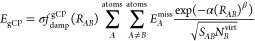
3where *A* and *B* denote atoms, σ is a global scaling parameter, *f*_damp_^gCP^ is a damping function as described in the work on the PBEh-3c^8^ method, α and β are global fit parameters, and *S*_*AB*_ is an s-type Slater overlap integral evaluated with scaled standard valence-average exponents. *E*_*A*_^miss^ is originally the atomic energy difference between a large, almost complete basis set and the target basis set, and *N*_*B*_^virt^ is the number of virtual orbitals in the target basis set. In r^2^SCAN-3c, both *E*_*A*_^miss^ and *N*_*B*_^virt^ are used as additional free fit parameters or are set to unity. In order to correct the remaining BSSE as well as the absorbing part of the (small) basis set incompleteness error (BSIE) in the STO-based r^2^SCAN-3c, the gCP correction was manually adjusted by optimizing the global scaling parameter σ after the basis set and the D4 correction had been modified. The remaining parameters are not altered. For the manual optimization of σ, mainly the S22, S66, and NCIBLIND10^44^ benchmark sets were analyzed to reduce overall deviations, but additional sets were also cross-checked. Figure 4 shows the impact of the gCP correction as well as the different global scaling factors for the WATER27 and ACONF12^12^ benchmark sets. Although individual test sets, such as WATER27, benefit from a large scaling factor close to one, the majority is better described with a smaller value (cf. Figure 4b), which indicates that the STO basis set mTZ2P is less prone to remaining basis set errors than the GTO basis set mTZVPP. A good balance for all tested benchmark sets is represented by the value σ = 0.879, which is applied instead of the originally used σ = 1.000.

### Relativistic Effects

In standard quantum chemical problems, the nonrelativistic Schrödinger equation is approximately solved to obtain the final wave function. However, relativistic effects can affect the molecular geometry as well as properties, especially when heavy atoms (typically with *Z* > 36) are present.^45−48^ They can be included implicitly by relativistic effective core-potentials (ECPs) that replace the core electrons. This approach has the advantage of a lower computational cost and is sufficient for most chemical problems which mainly depend on valence electrons like thermochemisty.^49^ Accordingly, the original GTO-based r^2^SCAN-3c is based on a modified Ahlrichs basis set that is constructed for default use with ECPs.

Relativistic effects can also be incorporated explicitly with the more time-consuming four-component Dirac equation that can be approximated with the zeroth-order regular approximation (ZORA).^30−32^ It is based on an expansion of the full relativistic Hamiltonian with respect to a potential-dependent perturbation parameter and contains relativistic corrections at the zeroth order.^32,50^ Overall, the accuracy for structures and electronic energies is typically similar to ECPs and ZORA.^51−53^ Nevertheless, ZORA is less approximate, and the application of an all-electron (AE) basis set gives the flexibility to also apply explicit spin–orbit relativistic Hamiltonians, which may be crucial for very heavy elements. Further, explicit description of the core electrons can be crucial for a correct description of properties such as NMR chemical shielding tensors. Since scalar-relativistic effects are typically dominant for most applications and ZORA is the default in the ADF program package, it is also applied in the STO-based r^2^SCAN-3c.

### Grid Study

**Figure 5:**
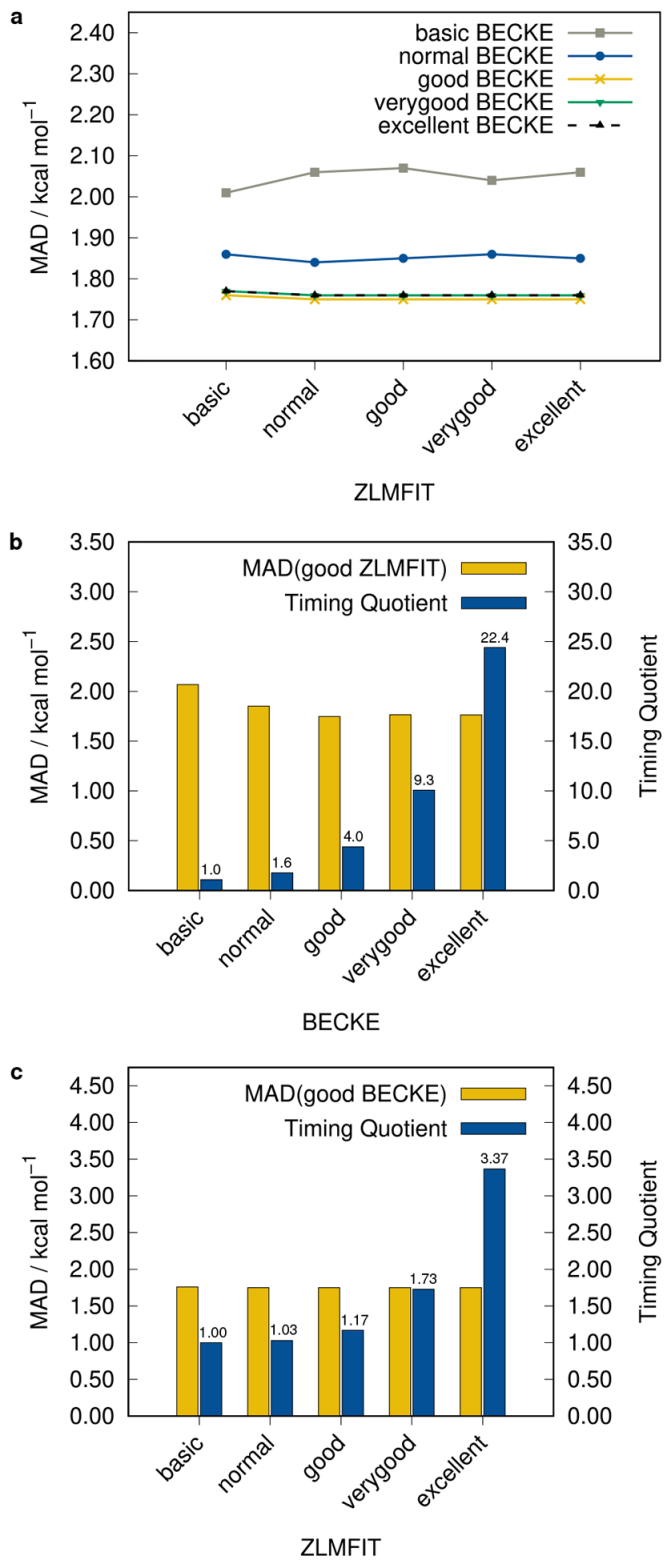
Mean absolute deviation (MAD) for the ACONF, ACONF12, L7, MOR41, S30L, and S22 benchmark sets calculated with r^2^SCAN-3c(STO): Different *BECKE* and *ZLMFIT* settings (a) as well as timing quotients relative to the *BECKE* setting *basic* (b) and relative to the *ZLMFIT* setting *basic* (c).

Any conventional DFT calculation is typically depending on a sufficiently fine numerical integration grid.^54^ And even though r^2^SCAN is already numerically more robust than its preceding functionals SCAN^18^ and rSCAN,^28^ the choice of a reasonable grid size is still relevant to obtain reliable results. In ADF, the grid can be controlled by the *NumericalQuality* setting, which simultaneously sets the quality of the *BECKE* integration grid and the quality of the density fitting, termed *ZLMFIT*. To determine a suitable default, different settings were assessed on a test set that includes the ACONF,^55^ ACONF12,^12^ L7, MOR41,^25^ S30L, and S22 benchmark sets. The performance of each *BECKE*/*ZLMFIT* combination as well as a timing comparison is depicted in Figure 5. It was found that the influence of *ZLMFIT* on the accuracy is negligible and that the results mainly depend on the *BECKE* grid, which is almost converged with the *good* setting. This goes along with an increased computational cost compared to the *normal* setting but is necessary in order to make the method robust. For the same reason, we decided to also set the *ZLMFIT* to *good* in all calculations. In general, the *good* setting for the *BECKE* grid leads to a slightly lower number of points on a Lebedev grid compared to the originally used *m4* angular grid in combination with a radial grid size of 10 in the GTO-based TURBOMOLE^56,57^ (TM) code (e.g., 460046 (ADF) vs 476590 (TM) for *n*-dodecane).

### Computational Details

Single-point calculations with r^2^SCAN-3c(STO) were performed with a development version of the Amsterdam Density Functional program ADF from the Amsterdam Modeling Suite AMS 2021.201 program package.^20,21^ Consideration of molecular symmetry was turned off and the *NumericalQuality* was set to *good*. Benchmark sets that require computations of single atoms (AHB21,^59^ ALKBDE10,^60^ BH76,^61−63^ BH76RC,^63^ CHB6,^59^ DIPCS10,^23^ G21EA,^63,64^ G21IP,^63,64^ HEAVYSB11,^23^ PA26,^23,60,63,65^ RG18,^23^ SIE4x4,^23^ W4-11^66^) were calculated with the *IntegerAufbau* option to obtain integer instead of fractional orbital occupations. Scalar-relativistic effects were treated with the zeroth-order regular approximation (ZORA). For the D4 London dispersion correction and the geometrical counterpoise (gCP) scheme, the standalone programs *dftd4 3.3.0*(13,14) and *mctc-gcp*(15) were used.

Further single-point calculations were conducted with PBE,^67^ TPSS,^68^ SCAN,^18^ r^2^SCAN,^16,17^ PBE0,^69,70^ and B3LYP^71,72^ in combination with the TZP, TZ2P, or QZ4P basis sets^73^ for a timing comparison. Additionally, geometry optimizations and single-point energies were calculated with BP86^74,75^-D4/TZP and M06-2X^76^-D3(0)^77,78^/TZP. The same settings as in the r^2^SCAN-3c calculations were used in all calculations.

Results for the GMTKN55 benchmark sets calculated with the GTO version of r^2^SCAN-3c were taken from the GMTKN55 database. The remaining test sets were computed with TURBOMOLE 7.5.1^56,57^ using r^2^SCAN-3c with grid m4 and a radial grid size of 10. The resolution of identity (RI) approximation for the Coulomb energy was used with the same reduced auxiliary basis sets developed originally for B97-3c.^79−81^ Default settings were used if not stated otherwise.

## Results and Discussion

To compare the STO-based composite r^2^SCAN-3c method with the original GTO-based approach, the performance of both implementations is assessed on eight geometry and 74 energy benchmark sets. The mean deviations (MDs), mean absolute deviations (MADs), standard deviations (SDs), and root-mean-square deviations (RMSDs) for each test set are listed in the Supporting Information.

### Geometries

**Figure 6:**
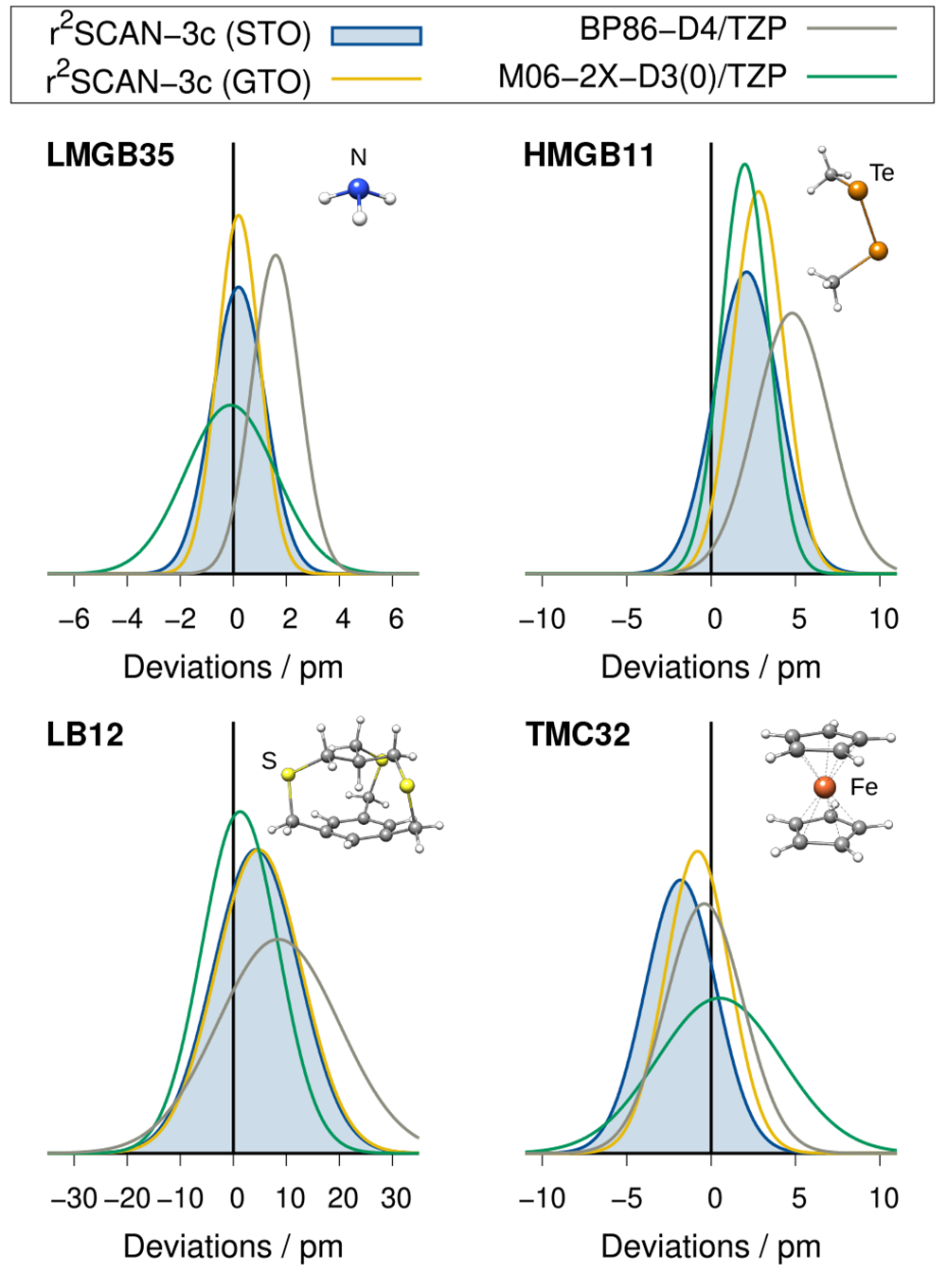
Gaussian error distributions for a selection of covalent bond length benchmark sets. Negative mean deviations indicate overall too short bond lengths.

**Figure 7:**
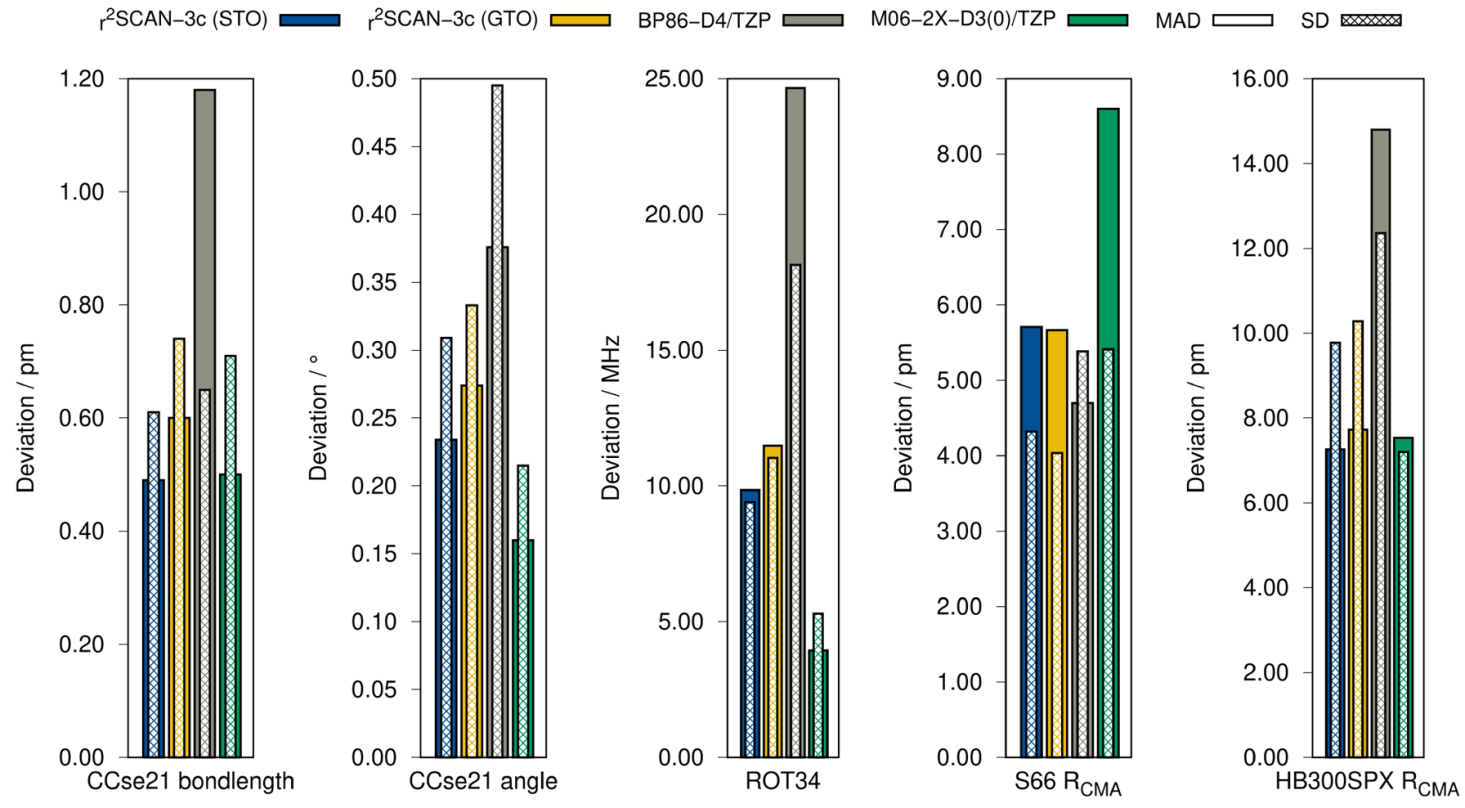
Mean absolute deviation (MAD) and standard deviation (SD) of different geometry benchmark sets calculated with both variants of r^2^SCAN-3c, as well as BP86-D4/TZP and M06-2X-D3(0)/TZP.

The performance for the calculation of covalent bond lengths is assessed on benchmark sets that contain light main-group bonds (LMGB35^82^), heavy main-group bonds (HMGB11^82^), long main-group bond lengths (LB12^82^), transition metal complexes (TMC32^83^), and small semirigid organic molecules (CCse21^84,85^). Rotational constants are evaluated with the ROT34^86,87^ test set. The results are summarized in Figures 6 and 7.

In general, the GTO- and STO-based approaches of r^2^SCAN-3c yield similar results for the molecular geometries. Both gravitate toward slightly too long bonds for light and heavy main group bonds and toward too short bonds for transition metal complexes. In general, the error spread of r^2^SCAN-3c(STO) tends to be slightly larger than that of the GTO-based method, as described by the standard deviation of each test set, but the difference is small and the mean absolute deviation is lower with the STO variant. Overall, it is either on par with or outperforms the commonly used GGA method BP86-D4/TZP as well as the meta-hybrid method M06-2X-D3(0)/TZP, which performs well for small organic molecules. In calculations of bond angles and rotational constants, r^2^SCAN-3c(STO) yields smaller errors than the GTO-based composite method (about 14% difference in the MAD for both cases).

To test noncovalent bonds, center-of-mass distances (*R*_CMA_) for the noncovalent interaction (NCI) benchmark sets S66x8^35,36,88^ and HB300SPXx10^37^ were calculated via a six-point cubic spline-interpolation ({0.90, 0.95, 1.00, 1.05, 1.10, 1.25}*R*_e_) of rigid fragment potential energy curves. The results are evaluated with respect to CCSD(T)/CBS and counterpoise-corrected MP2-F12/V{T,Q}Z-F12 reference values, respectively. The MADs and SDs are shown in Figure 7.

In the S66 test set, which contains organic van der Waals and hydrogen-bonded systems, both implementations of r^2^SCAN-3c perform equally well with very similar statistical measures. For example, both yield too long noncovalent bonds with a MD of 5.3 pm for the STO-based composite method and 5.4 pm for the GTO-based method. Im comparison, BP86-D4/TZP and M06-2X-D3(0)/TZP yield systematically too short NCI contacts with a MD of −2.5 and −8.6 pm, respectively. The performance of both variants of r^2^SCAN-3c is remarkable, as their SD is about 1 pm smaller than that of BP86/TZP and M06-2X and the MAD is about 2.9 pm smaller than that of M06-2X-D3(0)/TZP.

For the hydrogen-bonded systems in HB300SPX, r^2^SCAN-3c(STO) yields smaller deviations than the GTO-based method, and the respective MAD of 7.3 pm is halved compared to BP86/TZP, representing the smallest value in this study. Here, both versions of r^2^SCAN-3c yield slightly too short H-bonds with a MD of −1.5 pm for the STO variant and −0.8 pm for the GTO variant. These values are rather small compared to that of BP86-D4/TZP (MD = −12.9 pm) and M06-2X (MD = −6.6 pm), which both drastically underestimate H-bond lengths.

Overall, r^2^SCAN-3c(STO) yields very similar results as the GTO-based method and is, in most cases, even slightly more accurate. It reaches the accuracy of computationally much more demanding hybrid/TZ approaches and can therefore be recommended for geometry optimizations.

### Relative Energies

The study on relative energies includes 4405 data points in a range between −363.0 and 1290.7 kcal mol^–1^ with a mean reaction energy of 19.8 kcal mol^–1^ covering a broad area of the chemical space with tests for thermochemistry, reaction barriers, noncovalent interactions (NCIs), and conformational energies.

### Main-Group Thermochemistry and Reaction Barriers

**Figure 8:**
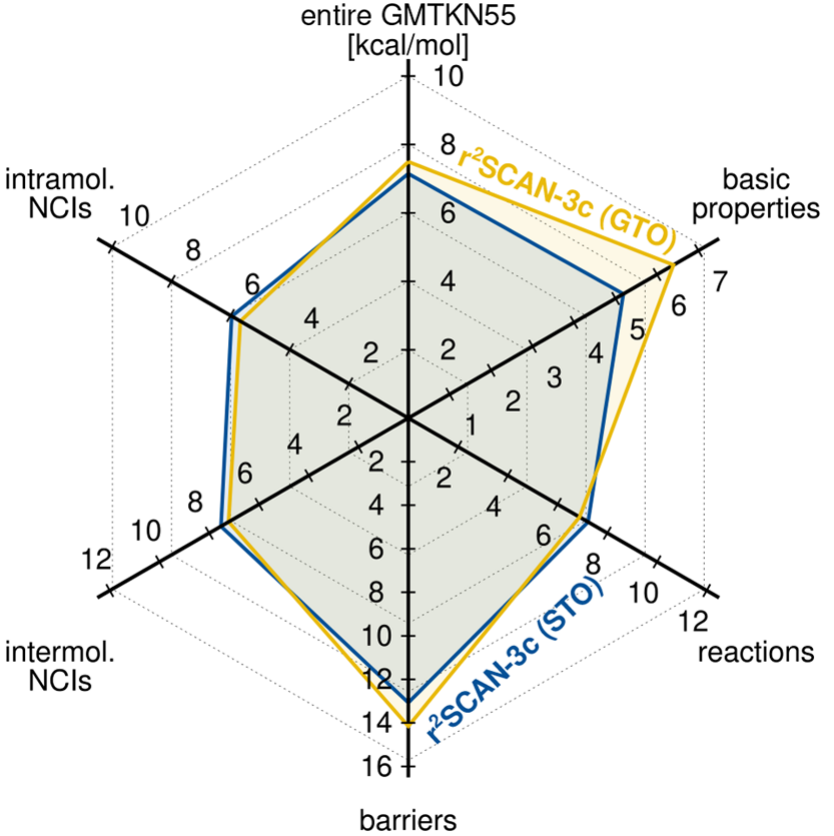
Weighted mean absolute deviation (WTMAD-2) of the GMTKN55 as well as its subclasses computed with both variants of r^2^SCAN-3c.

The extensive GMTKN55 database contains 55 versatile benchmark sets with CCSD(T)/CBS reference data for main-group thermochemistry, kinetics, and noncovalent interactions and represents an ideal base to validate density functional approximations. It consists of 1505 data points that can be categorized in the five subsets basic properties and reaction energies for small systems (basic properties), reaction energies for large systems (reactions), reaction barrier heights (barriers), and inter- as well as intramolecular NCIs. Because the average energies between the test sets vary significantly, the standard weighted MAD (WTMAD-2; see Supporting Information) is taken as a statistical performance measure.^23^ The results are shown in Figure 8 and Table 2.

**Table 2:** Weighted Mean Absolute Deviation (WTMAD-2) of the Entire GTMKN55, As Well As Its Subclasses Computed with r^2^SCAN-D4 and Both Variants of r^2^SCAN-3c

	TZP	TZ2P	QZ4P	3c(STO)	3c(GTO)
entire GMTKN55	8.45	7.97	7.41	7.15	7.50
basic properties	5.36	5.22	5.10	5.19	6.40
reactions	7.28	7.86	7.87	7.23	6.89
barriers	15.66	15.10	14.51	13.07	14.15
intermol. NCIs	9.84	9.03	7.33	7.53	7.22
intramol. NCIs	8.20	6.65	6.16	5.96	5.67

The STO-based r^2^SCAN-3c composite method surpasses the accuracy of the parent functional r^2^SCAN-D4 in nearly all categories, independent of the basis set size. This is especially noticeable in the WTMAD-2 of the entire GMTKN55 database, where the STO version of r^2^SCAN-3c (7.15 kcal mol^–1^) yields the best results, followed by r^2^SCAN-D4/QZ4P (7.41 kcal mol^–1^), with TZ2P (7.97 kcal mol^–1^) and TZP (8.45 kcal mol^–1^).

**Figure 9:**
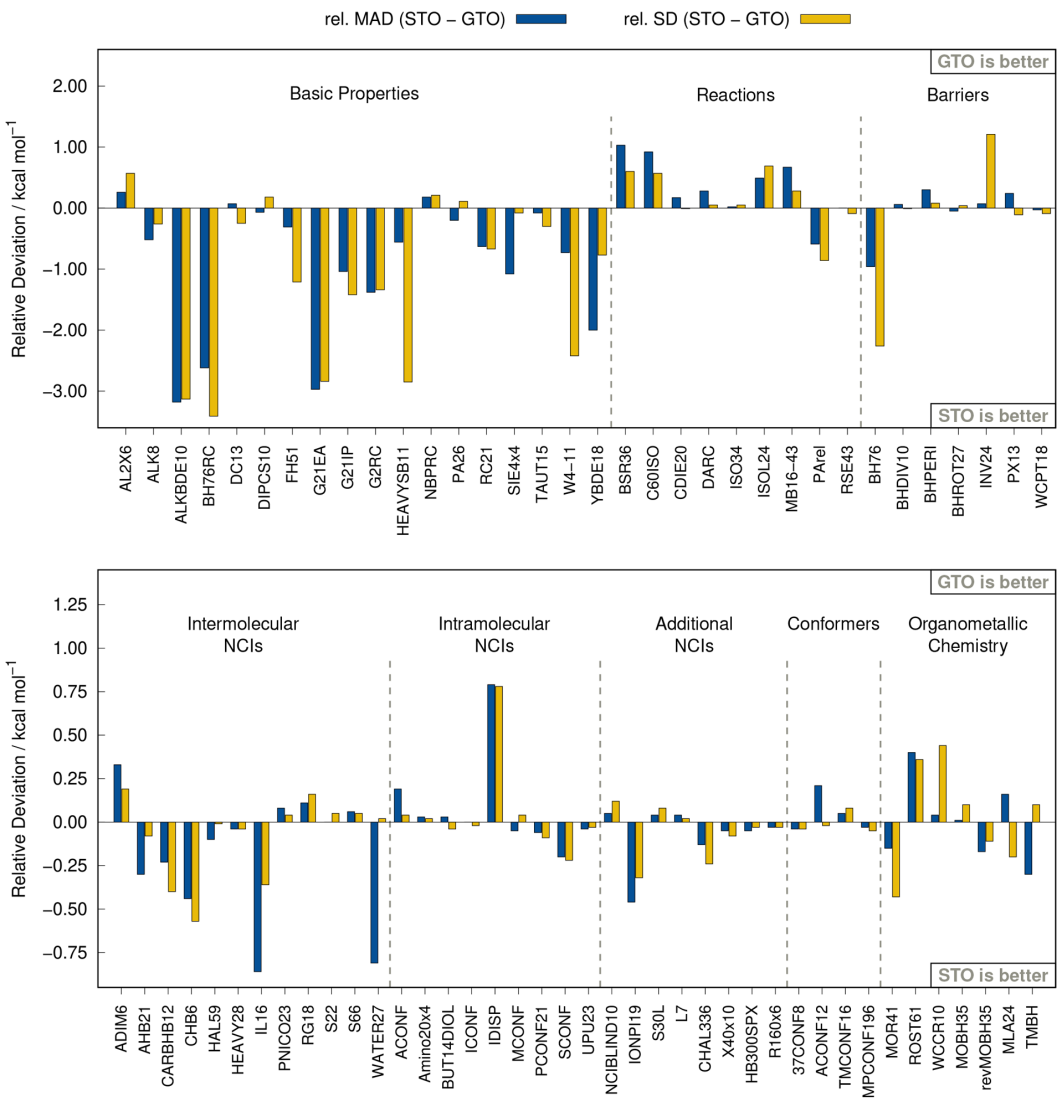
Relative mean absolute deviation (MAD) and standard deviation (SD) of the STO version of r^2^SCAN-3c with respect to r^2^SCAN-3c (GTO) calculated on the GMTKN55 and several other benchmark sets.

The WTMAD-2 of r^2^SCAN-3c(STO) for the entire database is also lower than that of the original GTO-based r^2^SCAN-3c (7.50 kcal mol^–1^). Notably, this performance approaches that of hybrid DFAs with large aug-def2-QZVP AO basis sets such as B3LYP-D4 (6.5 kcal mol^–1^) and PW6B95-D4 (5.5 kcal mol^–1^)^12^ with a drastically reduced computational cost. The overall accuracy of both r^2^SCAN-3c implementations is similar, but the GTO-based approach yields slightly more accurate results for reactions and intramolecular NCIs, while the STO-based approach yields better results for basic properties and barrier heights. This behavior is also depicted in Figure 9, which shows the difference in the MADs and SDs of both r^2^SCAN-3c implementations. In this comparison, every positive value represents a better description by the GTO version and every negative value a better description by the STO version of r^2^SCAN-3c. The good performance of r^2^SCAN-3c(STO) for the basic properties presumably stems from the larger basis set that seemingly reduces the self-interaction error (SIE), as is also observed in the SIE4x4 test set.

### Noncovalent Interactions

In addition to the GMTKN55 benchmark collection, more recent benchmark sets on noncovalent interactions are assessed in this section. Among them are large complexes (S30L, L7), various chalcogen (CHAL336^89^), halogen (X40x10^90^), and hydrogen bonding sets (HB300SPX), ion−π interactions (IONPI19), a blind test for DFT-based methods (NCIBLIND10), and repulsive intermolecular contacts (R160x6^12,91,92^). For the L7 set, average values of the respective LNO-CCSD(T) and fixed-node diffusion Monte Carlo (FN-DMC) interaction energies published by Al-Hamdani et al.^40^ were used as reference.

**Figure 10:**
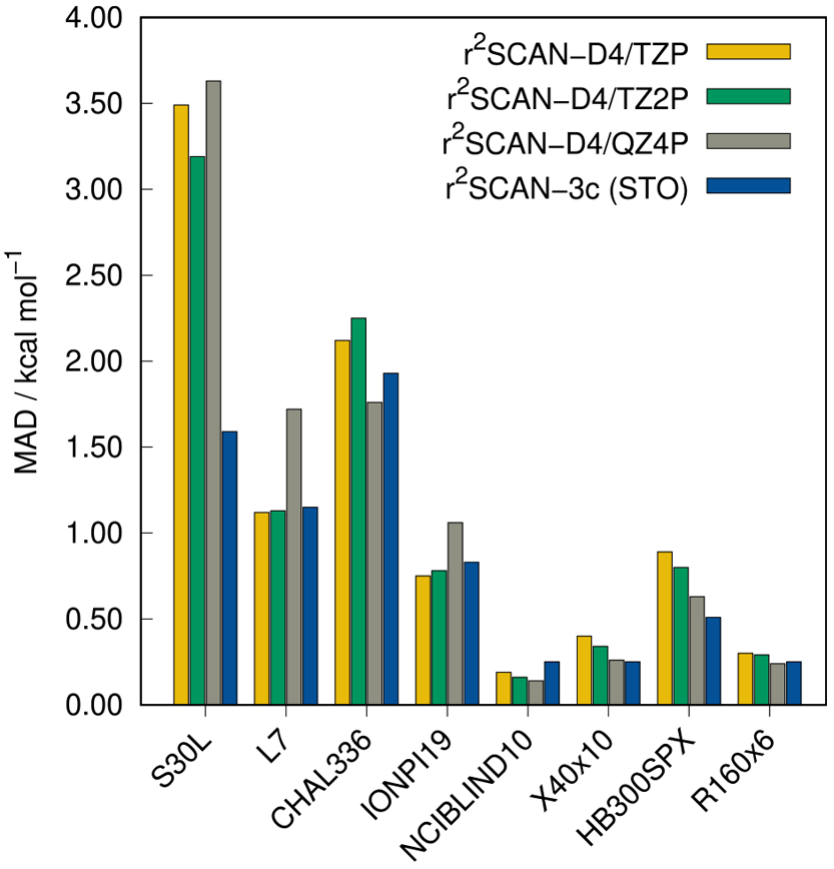
MADs of the additional NCI benchmark sets calculated with r^2^SCAN-3c(STO) and r^2^SCAN-D4 in combination with different STO basis sets.

**Figure 11:**
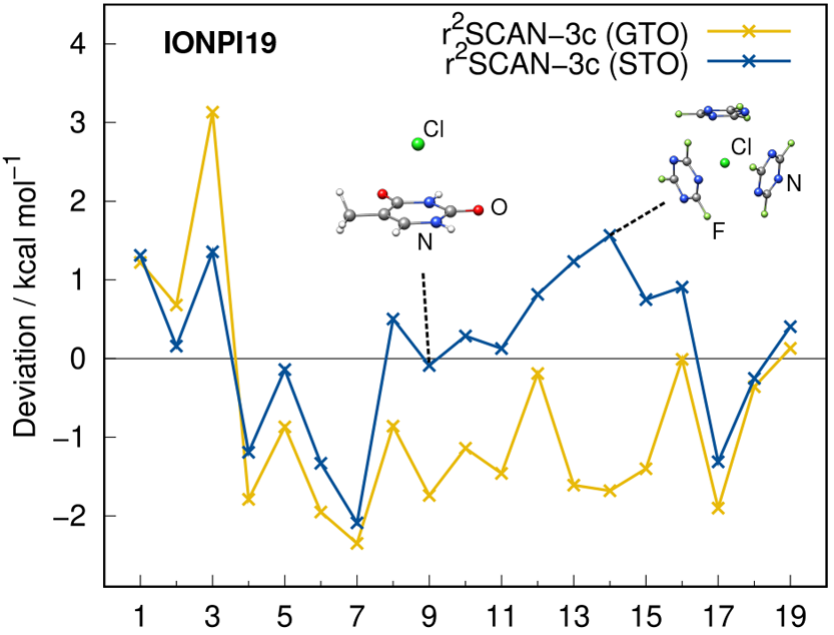
Deviations of the IONPI19 benchmark set calculated with both versions of r^2^SCAN-3c.

Similar to the statistics for the GMTKN55, the MAD values for the additional NCI benchmark sets in Figure 10 show that r^2^SCAN-3c(STO) is either on par with r^2^SCAN-D4/QZ4P or even more accurate. In particular, in the S30L benchmark for association energies of realistic host–guest complexes, r^2^SCAN-3c stands out as the MAD is 56% lower than that of r^2^SCAN-D4/QZ4P. The dominant contribution to the interaction energies is London dispersion, which might indicate that the parametrization of the D4 correction in r^2^SCAN-D4 is not optimal for STOs. Unexpectedly, it is also observed that the largest tested basis set, QZ4P, does not always yield more accurate results than the smaller basis sets. Compared to the original GTO-based r^2^SCAN-3c, the STO approach yields overall similar results (cf. Figure 9). The largest difference is observed in the IONPI19 benchmark set, which is shown in Figure 11. Here, the STO variant of r^2^SCAN-3c yields a 35% lower MAD of 0.83 kcal mol^–1^.

### Conformational Energies

The conformations of a molecule have a direct influence on chemical properties.^93,94^ Therefore, it can be crucial to consider a conformer ensemble that is routinely created with methods that apply semiempirical methods, for example, with the CREST algorithm.^95^ They still require a subsequent higher-level energy ranking for which DFT is usually employed.^96^ One of the remarkable features of the original r^2^SCAN-3c implementation is the very good performance for conformational energies where it surpasses the accuracy of hybrid-DFT/QZ approaches at a considerably lower computational cost.^12^ Thus, the implementation with STOs should ideally perform similarly.

**Figure 12:**
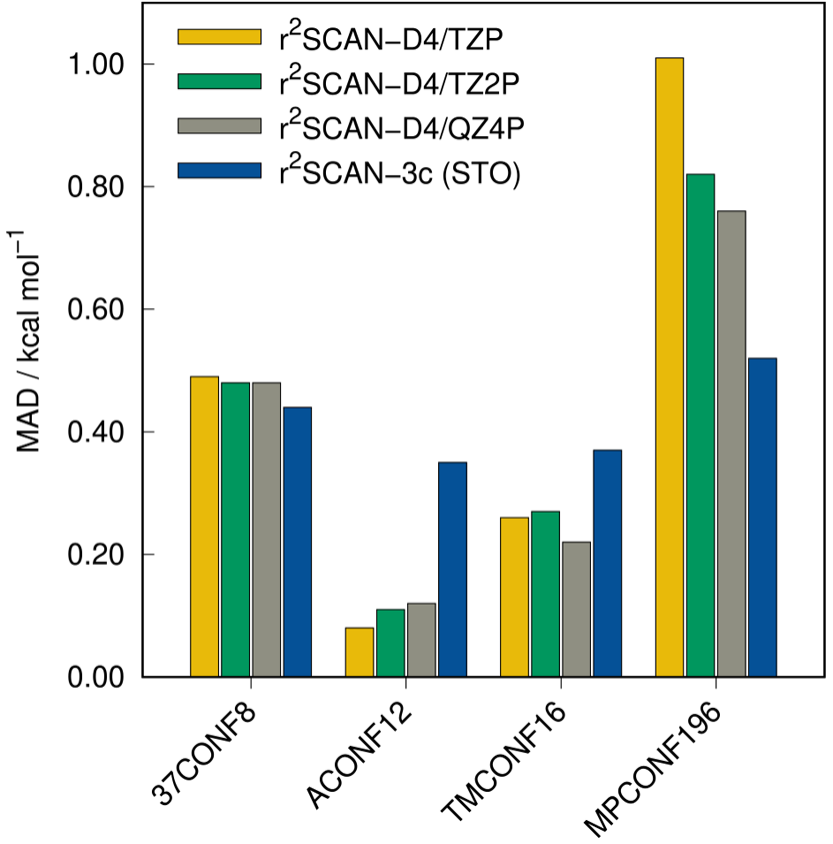
MADs of r^2^SCAN-3c(STO) and r^2^SCAN-D4 in combination with different STO basis sets for conformational energy benchmark sets.

**Figure 13:**
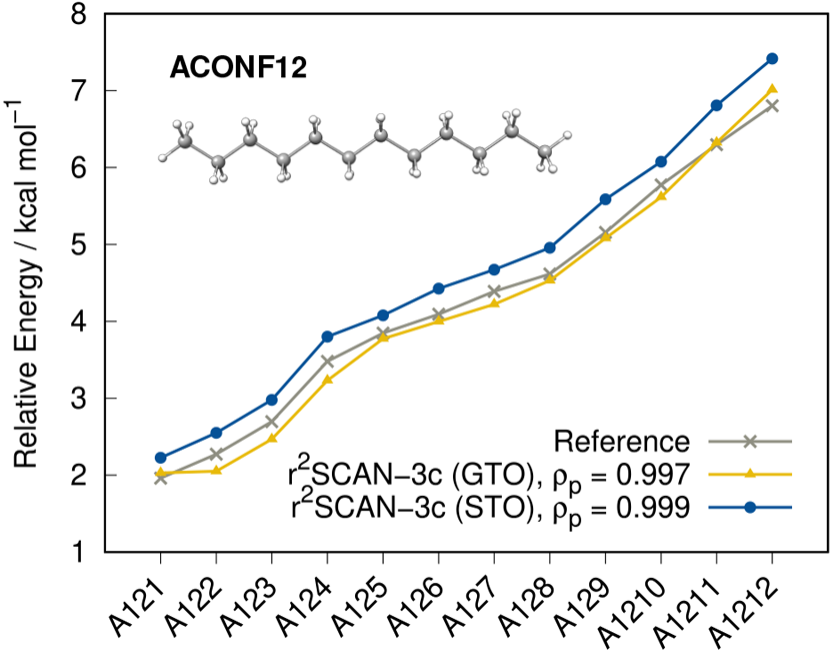
Conformational energies calculated with both versions of r^2^SCAN-3c for the ACONF12 benchmark set as well as the Pearson coefficients ρ_p_. The Spearman correlation coefficient is ρ_s_ = 1 for both variants of r^2^SCAN-3c. The reference was calculated at DLPNO–CCSD(T1)/*VeryTightPNO*/CBS level of theory.^12^

In addition to the eight conformer tests sets of the GMTKN55 database, the ACONF12 set with long alkane chains, TMCONF16^97^ with transition metal complexes, and MPCONF196,^98^ as well as 37CONF8,^99^ with large molecules are evaluated in this section. The TMCONF16 set is essentially the TMCONF5 benchmark set without the AYISEG system. The results are depicted in Figures 12 and 13.

For both large molecule test sets (37CONF8 and MPCONF196), r^2^SCAN-3c(STO) yields slightly better results than r^2^SCAN-D4, similar to the findings in the previous sections. However, it yields larger deviations for alkane chains and transition metal complexes. These deviations are also observed in the comparison between the results obtained with both implementations (Figure 9). Nevertheless, the error is still small and practically negligible. The conformational energies in the ACONF12 test set are depicted in Figure 13. While the MAD value of 0.14 kcal mol^–1^) with the GTO approach is lower than with the STOs (MAD = 0.35 kcal mol^–1^), the relative energy ranking is better described by r^2^SCAN-3c(STO), which can be derived from the better Pearson correlation coefficient (ρ_p_ = 0.999).

### Organometallic Thermochemistry

**Figure 14:**
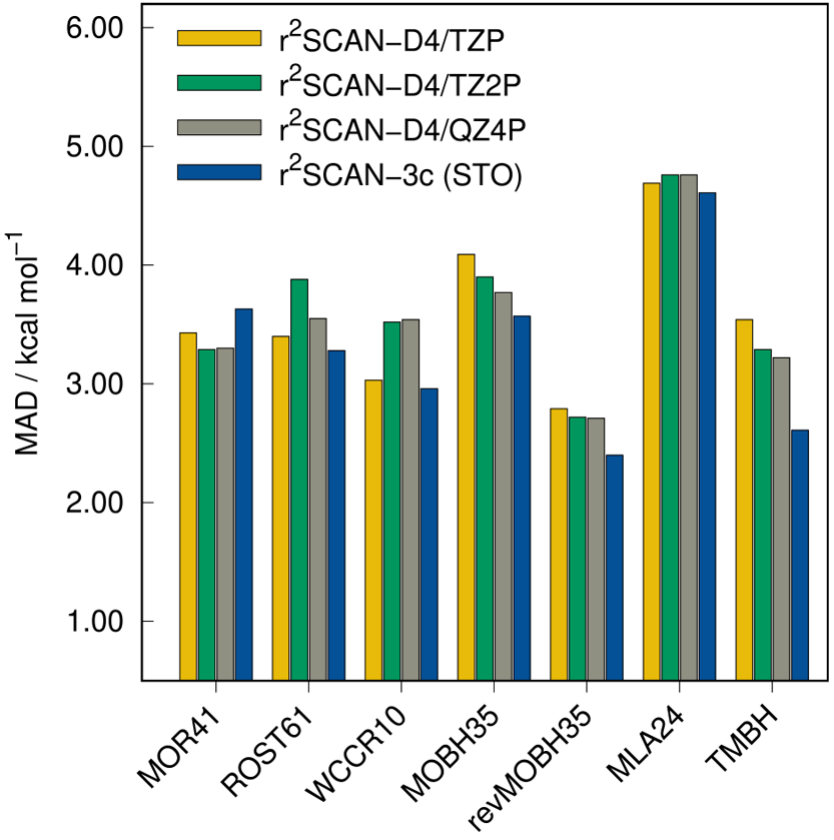
MADs of r^2^SCAN-3c(STO) and r^2^SCAN-D4 in combination with different STO basis sets for organometallic thermochemistry benchmark sets.

As the GMTKN55 database does not include any transition metal complexes, additional test sets are evaluated in this section. Reaction energies of closed-shell complexes are considered with the MOR41 and WCCR10^100,101^ benchmark sets, as well as open-shell systems in the ROST61^26^ set. Transition metal barrier heights are tested on the TMBH^102−105^ benchmark set, which contains 34 barrier heights and on the revised MOBH35 benchmark set, termed revMOBH35.^106^ The original MOBH35^107,108^ set is also included to provide comparability to prior works. Binding energies of metal-linked alkyl chains are assessed on the MLA24^109^ benchmark set. The results are depicted in Figures 9 and 14.

**Figure 15:**
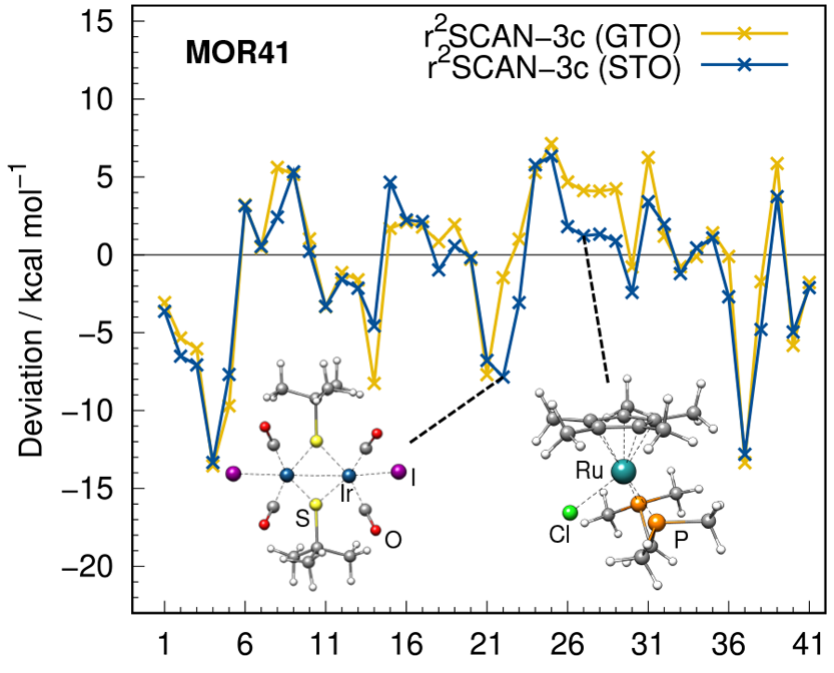
Deviations of the MOR41 benchmark set calculated with both versions of r^2^SCAN-3c.

In all tests except MOR41, r^2^SCAN-3c(STO) outperforms r^2^SCAN-D4, independent of the applied STO basis set. However, the differences between the DFAs are rather small considering the range of energies included. The largest difference is found for reaction barrier heights in the TMBH set where the MAD of r^2^SCAN-3c(STO) (MAD = 2.61 kcal mol^–1^) is 19% lower than the MAD of r^2^SCAN-D4/QZ4P (MAD = 2.91 kcal mol^–1^). Also, the deviation between both implementations of r^2^SCAN-3c is fairly small. While r^2^SCAN-3c(GTO) yields lower errors for open-shell systems (ROST61), the STO-based method yields lower errors for closed-shell systems (MOR41). The analysis for the MOR41 benchmark set in Figure 15 reveals that the STO-based approach is more accurate in this test set due to a better description of systems with π-interactions, which is in line with the findings for the noncovalent interaction benchmark set IONPI19 (cf. Figure 11).

### Computation Time

**Figure 16:**
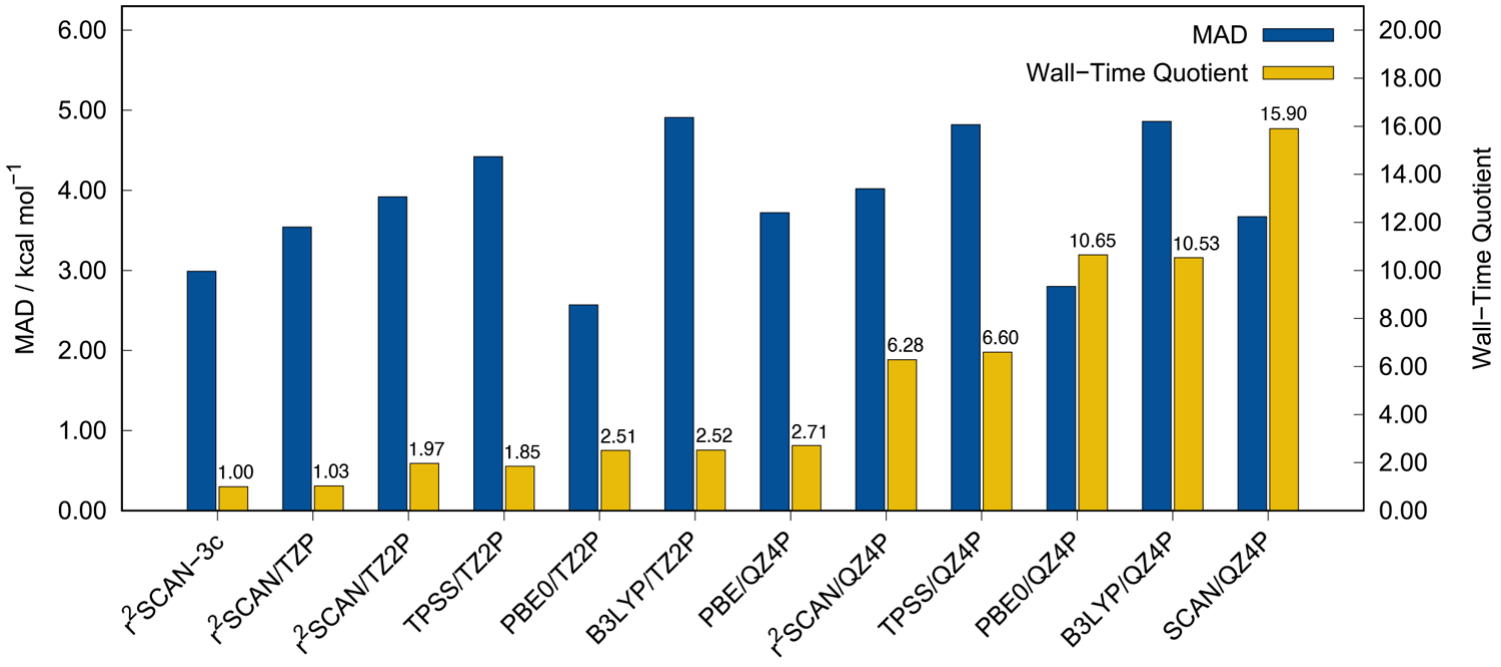
MADs and wall-time quotients relative to STO-type r^2^SCAN-3c (1.0 = 38691 s) in a single-point energy calculation for eight structures from the S30L, MOR41, L7, and ROST61 benchmark sets. All calculations were carried out with ADF and include the D4 correction. Note that the wall-times for the semiclassical D4 and gCP corrections are negligible at 0.35 and 0.15 s, respectively. Computations were done on four Intel Xeon CPU E3-1270 v5@3.60 GHz cores.

In this section, the timings for single-point energy calculations within the ADF code are assessed on a small test set that includes eight data points that were taken from the MOR41 (**13**, **40**), ROST61 (**R31**, **R33**), S30L (**9**, **19**), and L7 (**GGG**, **C2C2PD**) benchmark sets. The performance of all tested STO-based DFAs is depicted in Figure 16.

In this comparison, r^2^SCAN-3c(STO) is the most efficient DFA. It is faster than PBE0/TZ2P by a factor of 2.51 and faster than PBE0/QZ4P by a factor of 10.65. Surprisingly, the timing difference between meta-GGA and hybrid DFAs is not as large as in common GTO-based codes such as ORCA^110^ and TURBOMOLE,^56,57^ where hybrid DFAs are by a factor of about 15–20 slower than meta-GGA DFAs. In the STO-based ADF code, r^2^SCAN/TZ2P is only 1.3 times faster than PBE0/TZ2P, although the latter requires the additional computation of Fock exchange. This might be an effect of the Libxc implementation of r^2^SCAN(STO), which may slow down the computation. For example, the GGA PBE is about twice as fast in the native implementation compared to the Libxc variant (cf. Supporting Information).

**Figure 17:**
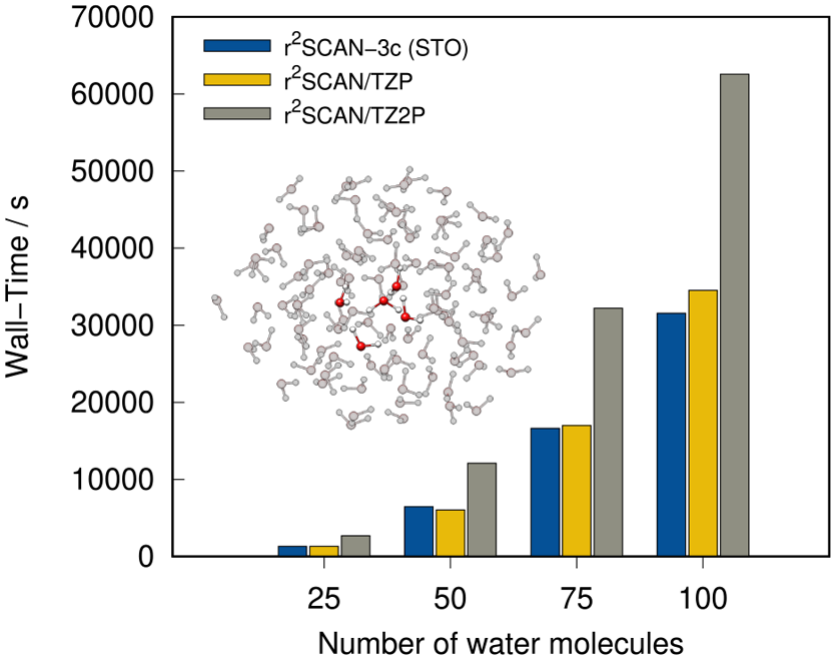
Wall-time for a single-point calculation of water clusters with different sizes applying r^2^SCAN-3c(STO) as well as r^2^SCAN-D4 in combination with TZP and TZ2P. All computations were done on four cores with the same CPU as in Figure 16.

To compare the modified basis set of r^2^SCAN-3c with the underlying TZP and TZ2P basis sets, we also tested the computation time on water clusters of different sizes. The results are depicted in Figure 17. Overall, the STO basis set applied in r^2^SCAN-3c lead to similar computation times as the TZP basis set and the mTZ2P basis set is about twice as fast as the TZ2P basis set.

## Conclusion

In this work, we presented the Slater-type atomic orbital basis set optimized variant of the composite electronic-structure method r^2^SCAN-3c. It combines the meta-generalized-gradient-approximation density functional r^2^SCAN with a tailored triple-ζ all-electron STO basis set and applies the readjusted semiclassical D4 and gCP corrections for London dispersion effects and basis set superposition errors, respectively. Instead of the originally applied effective core potentials in the GTO approach, relativistic effects are treated explicitly with the scalar-relativistic zeroth-order regular approximation (SR-ZORA), keeping the flexibility to also apply spin–orbit relativistic ZORA. For robust and accurate results, the *NumericalQuality* should be generally set to the *good* level in the ADF code.

In this comprehensive study, the performance of r^2^SCAN-3c was assessed on a collection of 82 benchmark sets that cover geometries, thermochemistry, barrier heights, noncovalent interactions, and conformational energies of main-group as well as transition metal systems. In total, 621 data points for geometrical properties and 4405 data points for energies were evaluated for both implementations of r^2^SCAN-3c. In the geometry study, r^2^SCAN-3c(STO) has proven to be on par with or better than the hybrid M06-2X-D3(0)/TZP approach. In the energy study, r^2^SCAN-3c was further compared to r^2^SCAN-D4 in combination with different sizes of STO basis sets. It was shown that r^2^SCAN-3c(STO), in most cases, provides more accurate results than r^2^SCAN-D4/QZ4P at a 6-fold speed-up. The most significant improvement over the large basis set was found for noncovalent interactions of large systems (S30L) where the MADs are 1.59 and 3.63 kcal mol^–1^, respectively. On average, r^2^SCAN-3c(STO) yields similar results as the original GTO version which was also observed for geometrical properties. Reaction energies and intramolecular NCIs, such as conformational energies, are slightly better described with the GTO approach but basic properties and intermolecular NCIs, such as ion-π interactions, are better described with the STO approach. This results in a lower WTMAD-2 for the GMTKN55 database with the STO version (WTMAD-2 = 7.15 kcal mol^–1^) instead of the GTO version (WTMAD-2 = 7.50 kcal mol^–1^). Overall, r^2^SCAN-3c reaches the accuracy of hybrid DFAs, which apply quadruple-ζ AO basis sets at a significantly reduced computational cost.

The fast, robust, and accurate STO-based r^2^SCAN-3c method can be applied safely for a broad range of quantum chemical problems and therefore represents an efficient choice in many chemical applications.

## Supporting Information Available

D4 parameters for both variants of r^2^SCAN-3c, statistical measures for the benchmark sets, and timings (PDF)
