# Supplementary material for: Optimization of the r2SCAN-3c Composite Electronic-Structure Method for Use with Slater-Type Orbital Basis Sets
Source: J Phys Chem A. 2022 Jun 2;126(23):3826–38. doi: 10.1021/acs.jpca.2c02951 (PMC9255700; doi:10.1021/acs.jpca.2c02951)
Supplement: Supplementary file 1 — jp2c02951_si_001.pdf [file jp2c02951_si_001.pdf]

Supporting Information

Optimization of the r²SCAN-3c Composite Electronic-Structure Method for Use with Slater-Type Orbital Basis Sets

Thomas Gasevic,[†] Julius B. Stückrath,[†] Stefan Grimme,[*,†] and Markus Bursch[*,‡]

†*Mulliken Center for Theoretical Chemistry, Universität Bonn, Beringstr. 4, D-53115 Bonn, Germany*

‡*Max-Planck-Institut für Kohlenforschung, Kaiser-Wilhelm-Platz 1, D-45470 Mülheim an der Ruhr, Germany*

E-mail: grimme@thch.uni-bonn.de; bursch@kofo.mpg.de

Contents

S1 Utilized D4 Parameters **S3**

S2 Statistical Quantities & Benchmark Sets **S3**

S2.1 Geometry Benchmark Sets S5

S2.2 Energy Benchmark Sets S7

S3 Timings **S14**

S1 Utilized D4 Parameters

Table S1: D4 parameters for the GTO and STO variants of r^{2}SCAN-3c. The change in the STO approach is marked in bold.

	GTO mTZVPP	STO mTZ2P
s_6	1.00	1.00
s_8	0.00	0.00
s_9	2.00	**1.53**
a_1	0.42	0.42
a_2	5.65	5.65
β	2.00	2.00
γ	1.00	1.00

S2 Statistical Quantities & Benchmark Sets

- Mean deviation (MD):

$$MD = \frac{1}{N}\sum_i (x_i - r_i) \tag{1}$$

- Mean absolute deviation:

$$MAD = \frac{1}{N}\sum_i |x_i - r_i| \tag{2}$$

- Standard Deviation:

$$SD = \sqrt{\frac{\sum_i^n |(x_i - r_i) - MD|^2}{N-1}} \tag{3}$$

- Root mean square deviation:

$$RMSD = \sqrt{\frac{1}{N}\sum_{i=1}^{N}(x_i - r_i)^2} \tag{4}$$

- Weighted mean absolute deviation:

$$WTMAD-2 = \frac{1}{\sum_i^n N_i} \sum_i^n N_i \cdot \frac{57.82\,\text{kcal mol}^{-1}}{\overline{|\Delta E|}_i} \cdot \text{MAD}_i \tag{5}$$

with N being the number of data points, x_i the calculated values, r_i the reference values, n the number of test sets, and $\overline{|\Delta E|}$ the mean absolute reference energy of a test set. The value of 57.82 kcal mol^{-1} represents the average over all mean absolute reference energies in the GTMKN55 database.

S2.1 Geometry Benchmark Sets

Table S2: Statistical measures for the deviation of the bond lengths in the LMGB35 benchmark set.

**LMGB35: Δ (bond length) / pm**				
	MD	MAD	SD	AMAX
r^2SCAN-3c (STO)	0.18	0.63	0.96	4.21
r^2SCAN-3c (GTO)	0.19	0.59	0.77	2.48
BP86-D4/TZP	1.59	1.59	0.86	4.56
M06-2X-B3(0)/TZP	−0.12	0.96	1.68	7.55

Table S3: Statistical measures for the deviation of the bond lengths in the HMGB11 benchmark set.

**HMGB35: Δ (bond length) / pm**				
	MD	MAD	SD	AMAX
r^2SCAN-3c (STO)	2.08	2.18	1.91	4.66
r^2SCAN-3c (GTO)	2.80	2.80	1.47	4.30
BP86-D4/TZP	4.84	4.84	2.16	8.11
M06-2X-B3(0)/TZP	1.97	2.00	1.38	4.55

Table S4: Statistical measures for the deviation of the bond lengths in the LB12 benchmark set.

**LB12: Δ (bond length) / pm**				
	MD	MAD	SD	AMAX
r^2SCAN-3c (STO)	4.33	5.97	8.08	23.98
r^2SCAN-3c (GTO)	4.83	6.15	8.13	25.40
BP86-D4/TZP	8.51	9.62	11.49	37.59
M06-2X-B3(0)/TZP	1.32	5.26	7.23	13.45

Table S5: Statistical measures for the deviation of the bond lengths in the TMC32 benchmark set.

**TMC32: Δ (bond length) / pm**				
	MD	MAD	SD	AMAX
r^2SCAN-3c (STO)	−1.84	2.25	2.14	7.60
r^2SCAN-3c (GTO)	−0.78	1.63	1.92	6.30
BP86-D4/TZP	−0.42	1.78	2.27	5.97
M06-2X-B3(0)/TZP	0.51	2.97	3.67	8.01

Table S6: Statistical measures for the deviation of the bond lengths in the CCse21 benchmark set.

**CCse21: Δ (bond length) / pm**				
	MD	MAD	SD	AMAX
r^2SCAN-3c (STO)	0.25	0.49	0.49	2.10
r^2SCAN-3c (GTO)	0.39	0.60	0.74	3.99
BP86-D4/TZP	1.12	1.18	0.65	3.51
M06-2X-B3(0)/TZP	−0.01	0.50	0.71	3.66

Table S7: Statistical measures for the deviation of the bond angles as well as the RMSD of the overall structure in the CCse21 benchmark set.

	**CCse21: Δ (angle) / °**				**Structure**
	MD	MAD	SD	AMAX	RMSD (Å)
r^2SCAN-3c (STO)	-0.01	0.23	0.31	0.92	0.56
r^2SCAN-3c (GTO)	-0.03	0.27	0.33	1.00	0.66
BP86-D4/TZP	-0.04	0.38	0.49	1.15	1.27
M06-2X-B3(0)/TZP	0.03	0.16	0.21	0.60	0.45

Table S8: Statistical measures for the deviation of the rotational constants in the ROT34 benchmark set.

**ROT34: Δ (rot. constant) / MHz**				
	MD	MAD	SD	AMAX
r^2SCAN-3c (STO)	−9.66	9.84	9.40	36.70
r^2SCAN-3c (GTO)	−10.86	11.48	11.03	44.00
BP86-D4/TZP	−24.65	24.65	18.14	85.80
M06-2X-B3(0)/TZP	−1.86	3.94	5.30	17.60

Table S9: Statistical measures for the deviation of the center of mass (CMA) distances obtained from spline-interpolated rigid fragment potential energy curves using 6 data points.

S66: Δ (R_{CMA}) / pm

	MD	MAD	SD	AMAX
r^2SCAN-3c (STO)	5.33	5.71	4.32	15.78
r^2SCAN-3c (GTO)	5.42	5.67	4.04	14.85
BP86-D4/TZP	-2.48	4.70	5.39	18.62
M06-2X-B3(0)/TZP	-8.60	8.60	5.41	22.02

HB300SPX: Δ (R_{CMA}) / pm

	MD	MAD	SD	AMAX
r^2SCAN-3c (STO)	-1.46	7.26	9.77	49.30
r^2SCAN-3c (GTO)	-0.79	7.73	10.28	44.71
BP86-D4/TZP	-12.89	14.79	12.36	63.60
M06-2X-B3(0)/TZP	-6.58	7.53	7.20	64.75

S2.2 Energy Benchmark Sets

Table S10: Number of data points N, average of the absolute energies $\overline{|E|}$ and mean deviations (MD) in kcal mol^1 for each test set computed with r^2SCAN in combination with different basis sets. The respective D4 London dispersion correction was applied in all calculations.

			MD				
Set	N	$\overline{\|E\|}$	3c(STO)	3c(GTO)	TZP	TZ2P	QZ4P
AL2X6	6	35.88	0.13	0.92	0.88	1.35	1.36
ALK8	8	62.60	1.96	3.05	2.04	2.12	2.14
ALKBDE10	10	100.69	0.54	−2.73	0.72	1.62	2.22
BH76RC	30	21.39	−0.50	−1.83	−0.14	−0.14	−0.01
DC13	13	54.98	1.45	3.02	1.10	1.25	1.46
DIPCS10	10	654.26	−1.61	−2.86	−0.69	−1.41	−2.59
FH51	51	31.01	0.08	0.34	−0.11	−0.09	−0.19
G21EA	25	33.62	−3.78	−5.86	−3.16	−3.44	−1.19
G21IP	36	257.61	−0.19	0.67	0.19	−0.03	−0.35
G2RC	25	51.26	0.19	−1.48	−0.99	−0.62	−0.59
HEAVYSB11	11	58.02	−4.32	−2.42	−4.22	−3.40	−3.64
NBPRC	12	27.71	0.99	0.99	−2.03	−0.56	−0.87
PA26	26	189.05	0.71	1.17	0.80	1.28	2.44
RC21	21	35.70	4.81	5.33	5.67	4.84	5.03
SIE4x4	16	33.73	16.68	17.76	17.19	17.54	17.95
TAUT15	15	3.05	−0.26	−0.03	0.71	0.33	0.29
W4-11	140	306.91	−3.12	−2.91	−4.09	−1.11	0.78
YBDE18	18	49.28	−5.07	−7.00	−4.69	−3.93	−3.08
BSR36	36	16.20	−1.35	−0.32	−0.36	−0.18	0.17
C60ISO	9	98.25	−5.95	−5.00	−5.86	−4.93	−5.47

CDIE20	20	4.06	1.30	1.13	1.50	1.64	1.64
DARC	14	32.47	0.41	−0.34	−0.56	1.62	1.07
ISO34	34	14.57	0.20	0.12	−0.08	−0.06	−0.11
ISOL24	24	21.92	−0.88	−0.59	−0.63	−1.06	−0.85
MB16-43	43	468.39	2.22	2.06	1.78	5.88	9.66
PArel	20	4.63	0.35	0.22	0.30	0.55	0.66
RSE43	43	7.60	−1.14	−1.10	−1.13	−1.31	−1.46
BH76	76	18.61	−6.80	−6.72	−8.03	−7.80	−7.34
BHDIV10	10	45.33	−4.33	−4.21	−5.33	−5.33	−5.13
BHPERI	26	20.87	−5.01	−4.71	−5.38	−4.87	−4.69
BHROT27	27	6.27	0.52	0.60	0.52	0.66	0.73
INV24	24	31.85	0.39	0.82	0.49	0.16	−1.72
PX13	13	33.36	−6.75	−6.51	−10.84	−9.69	−8.97
WCPT18	18	34.99	−4.38	−4.41	−7.10	−6.45	−6.18
ADIM6	6	3.36	−0.48	−0.15	−0.20	−0.16	−0.18
AHB21	21	22.49	−2.25	−2.49	−3.58	−3.43	−2.27
CARBHB12	12	6.04	1.35	1.58	1.52	1.40	1.12
CHB6	6	26.79	0.67	0.98	1.43	1.30	0.09
HAL59	59	4.59	0.86	0.95	1.01	0.93	0.74
HEAVY28	28	1.24	−0.12	−0.16	−0.22	−0.13	−0.22
IL16	16	109.05	−0.07	−1.61	−2.20	−2.13	−1.13
PNICO23	23	4.27	0.76	0.69	0.83	0.85	0.74
RG18	18	0.58	0.02	0.02	0.19	0.19	0.10
S22	22	7.30	−0.12	0.05	0.53	0.42	0.36
S66	66	5.47	−0.05	0.05	0.45	0.37	0.27
WATER27	27	81.17	3.71	4.57	12.34	10.22	6.94
ACONF	15	1.83	0.26	0.00	0.17	0.19	0.26
Amino20x4	80	2.44	−0.07	−0.02	0.07	0.04	0.03
BUT14DIOL	64	2.80	−0.18	−0.10	0.56	0.36	0.25
ICONF	17	3.27	0.05	−0.03	0.30	0.21	0.24
IDISP	6	14.22	2.09	1.73	1.46	1.70	1.53
MCONF	51	4.97	0.38	0.47	0.54	0.42	0.39
PCONF21	18	1.62	−0.04	−0.21	−0.31	−0.18	−0.21
SCONF	17	4.60	0.00	0.14	0.65	0.45	0.34
UPU23	23	5.72	−0.01	−0.05	−0.09	−0.15	−0.35
NCIBLIND10	80	2.54	0.09	0.03	−0.15	−0.11	−0.09
IONPI19	19	20.87	0.16	−0.74	0.04	0.00	0.09
S30L	30	37.51	−0.57	−1.02	−3.49	−3.19	−3.63
L7	7	16.21	0.50	−0.17	−0.56	−0.61	−1.25
CHAL336	336	14.09	−1.37	−1.87	−1.74	−2.10	−1.56
X40x10	400	2.71	−0.05	−0.09	−0.31	−0.26	−0.16
HB300SPX	300	4.58	−0.39	−0.48	−0.87	−0.78	−0.59
R160x6	960	2.04	0.08	0.11	−0.22	−0.20	−0.03
37CONF8	259	5.52	−0.08	−0.15	0.17	0.12	0.08
ACONF12	12	4.28	0.35	−0.09	0.03	0.08	0.08
TMCONF5	16	3.15	−0.15	−0.13	−0.05	−0.07	−0.07
MPCONF196	183	8.19	−0.01	0.06	0.75	0.57	0.48
MOR41	41	31.18	−1.23	−0.41	−1.91	−1.41	−0.59
ROST61	61	42.78	0.75	0.28	0.43	0.72	0.92
WCCR10	10	48.71	0.65	1.16	2.29	2.41	2.33
MOBH35	70	21.39	−2.66	−2.58	−3.26	−3.10	−2.98
revMOBH35	58	20.48	0.32	0.36	0.09	0.14	0.26
MLA	24	48.82	−1.85	−1.79	−2.32	−2.14	−2.09
TMBH	34	14.01	1.57	0.89	1.38	1.10	1.02

Table S11: Number of data points N, average of the absolute energies $\overline{|E|}$ and mean absolute deviations (MAD) in kcal mol^{1} for each test set computed with r^{2}SCAN in combination with different basis sets. The respective D4 London dispersion correction was applied in all calculations.

			MAD				
Set	N	$\overline{\lvert E \rvert}$	3c(STO)	3c(GTO)	TZP	TZ2P	QZ4P
AL2X6	6	35.88	1.20	0.94	1.34	1.52	1.36
ALK8	8	62.60	2.81	3.33	2.91	2.93	2.99
ALKBDE10	10	100.69	4.88	8.06	4.94	5.16	5.03
BH76RC	30	21.39	3.30	5.92	2.83	2.99	3.15
DC13	13	54.98	8.47	8.40	7.90	8.92	7.67
DIPCS10	10	654.26	4.70	4.77	5.19	4.81	4.92
FH51	51	31.01	2.47	2.78	2.46	2.19	2.04
G21EA	25	33.62	5.66	8.63	5.41	5.36	4.22
G21IP	36	257.61	4.52	5.56	4.33	4.46	4.67
G2RC	25	51.26	5.52	6.90	5.45	5.41	5.23
HEAVYSB11	11	58.02	4.32	4.88	4.22	3.46	3.64
NBPRC	12	27.71	1.96	1.78	3.44	1.46	1.57
PA26	26	189.05	1.72	1.92	1.79	1.74	2.47
RC21	21	35.70	5.26	5.89	6.09	5.29	5.55
SIE4x4	16	33.73	16.68	17.76	17.19	17.54	17.95
TAUT15	15	3.05	1.24	1.32	1.40	1.65	1.65
W4-11	140	306.91	4.62	5.35	5.22	3.66	3.75
YBDE18	18	49.28	5.38	7.38	5.32	4.44	3.53
BSR36	36	16.20	1.35	0.32	0.38	0.28	0.27
C60ISO	9	98.25	6.03	5.11	5.94	5.15	5.61
CDIE20	20	4.06	1.30	1.13	1.50	1.64	1.64
DARC	14	32.47	1.90	1.62	1.55	2.33	2.08
ISO34	34	14.57	1.39	1.37	1.20	1.34	1.23
ISOL24	24	21.92	3.28	2.79	3.26	4.04	3.57
MB16-43	43	468.39	12.75	12.08	12.04	13.69	14.43
PArel	20	4.63	1.18	1.77	1.66	1.53	1.53
RSE43	43	7.60	1.15	1.15	1.15	1.31	1.46
BH76	76	18.61	6.84	7.80	8.07	7.82	7.39
BHDIV10	10	45.33	5.25	5.19	6.26	6.26	6.04
BHPERI	26	20.87	5.01	4.71	5.38	4.87	4.69
BHROT27	27	6.27	0.55	0.60	0.57	0.69	0.73
INV24	24	31.85	1.71	1.64	1.60	1.65	2.00
PX13	13	33.36	6.75	6.51	10.84	9.69	8.97
WCPT18	18	34.99	4.38	4.41	7.10	6.45	6.18
ADIM6	6	3.36	0.48	0.15	0.20	0.16	0.18
AHB21	21	22.49	2.28	2.58	3.58	3.43	2.27
CARBHB12	12	6.04	1.35	1.58	1.52	1.40	1.12
CHB6	6	26.79	0.70	1.14	1.43	1.30	0.43
HAL59	59	4.59	0.95	1.05	1.09	0.98	0.85
HEAVY28	28	1.24	0.24	0.28	0.30	0.26	0.27
IL16	16	109.05	0.80	1.66	2.20	2.13	1.13
PNICO23	23	4.27	0.79	0.71	0.84	0.87	0.78
RG18	18	0.58	0.21	0.10	0.24	0.25	0.16
S22	22	7.30	0.34	0.34	0.53	0.44	0.37
S66	66	5.47	0.25	0.19	0.48	0.40	0.32
WATER27	27	81.17	3.97	4.78	12.90	10.74	7.40
ACONF	15	1.83	0.26	0.07	0.17	0.19	0.26

Amino20x4	80	2.44	0.23	0.20	0.21	0.18	0.18
BUT14DIOL	64	2.80	0.20	0.17	0.56	0.37	0.26
ICONF	17	3.27	0.36	0.36	0.40	0.32	0.29
IDISP	6	14.22	2.80	2.01	2.02	2.42	1.96
MCONF	51	4.97	0.44	0.49	0.57	0.51	0.49
PCONF21	18	1.62	0.54	0.60	0.54	0.49	0.53
SCONF	17	4.60	0.15	0.35	1.11	0.73	0.53
UPU23	23	5.72	0.41	0.45	0.34	0.35	0.46
NCIBLIND10	80	2.54	0.25	0.21	0.19	0.16	0.14
IONPI19	19	20.87	0.83	1.29	0.75	0.78	1.06
S30L	30	37.51	1.59	1.54	3.49	3.19	3.63
L7	7	16.21	1.15	1.12	1.12	1.13	1.72
CHAL336	336	14.09	1.93	2.06	2.12	2.25	1.76
X40x10	400	2.71	0.25	0.30	0.40	0.34	0.26
HB300SPX	300	4.58	0.51	0.56	0.89	0.80	0.63
R160x6	960	2.04	0.25	0.28	0.30	0.29	0.24
37CONF8	259	5.52	0.44	0.47	0.49	0.48	0.48
ACONF12	12	4.28	0.35	0.14	0.08	0.11	0.12
TMCONF5	16	3.15	0.37	0.32	0.26	0.27	0.22
MPCONF196	183	8.19	0.52	0.55	1.01	0.82	0.76
MOR41	41	31.18	3.63	3.78	3.43	3.29	3.30
ROST61	61	42.78	3.28	2.89	3.40	3.88	3.55
WCCR10	10	48.71	2.96	2.92	3.03	3.52	3.54
MOBH35	70	21.39	3.57	3.56	4.09	3.90	3.77
revMOBH35	58	20.48	2.40	2.57	2.79	2.72	2.71
MLA	24	48.82	4.61	4.45	4.69	4.76	4.76
TMBH	34	14.01	2.61	2.91	3.54	3.29	3.22

Table S12: Number of data points N, average of the absolute energies $\overline{|E|}$ and standard deviations (SD) in kcal mol[1] for each test set computed with r^{2}SCAN in combination with different basis sets. The respective D4 London dispersion correction was applied in all calculations.

			SD				
Set	N	$\overline{\lvert E\rvert}$	3c(STO)	3c(GTO)	TZP	TZ2P	QZ4P
AL2X6	6	35.88	1.36	0.80	1.26	1.22	1.04
ALK8	8	62.60	3.46	3.71	3.50	3.49	3.58
ALKBDE10	10	100.69	6.52	9.65	6.64	7.48	7.50
BH76RC	30	21.39	4.14	7.56	3.96	4.15	4.22
DC13	13	54.98	12.14	12.39	11.46	12.10	11.38
DIPCS10	10	654.26	5.71	5.52	6.19	5.81	5.50
FH51	51	31.01	3.53	4.74	3.72	3.44	3.33
G21EA	25	33.62	5.84	8.68	5.62	5.43	4.68
G21IP	36	257.61	5.55	6.97	5.55	5.54	5.75
G2RC	25	51.26	6.94	8.28	7.04	6.83	6.56
HEAVYSB11	11	58.02	1.99	4.84	1.97	2.45	2.57
NBPRC	12	27.71	2.33	2.11	3.45	1.90	1.95
PA26	26	189.05	2.56	2.45	2.66	2.21	2.18
RC21	21	35.70	4.75	5.43	5.21	5.00	5.11
SIE4x4	16	33.73	10.76	10.85	10.75	10.59	10.27
TAUT15	15	3.05	1.48	1.78	1.82	2.11	2.22
W4-11	140	306.91	4.92	7.34	4.90	4.87	5.10
YBDE18	18	49.28	2.75	3.52	3.11	2.74	2.53

BSR36	36	16.20	0.83	0.23	0.42	0.38	0.29
C60ISO	9	98.25	5.72	5.14	5.66	5.31	5.60
CDIE20	20	4.06	0.40	0.40	0.49	0.61	0.66
DARC	14	32.47	2.19	2.14	2.00	2.15	2.17
ISO34	34	14.57	1.99	1.95	1.98	2.15	1.98
ISOL24	24	21.92	4.96	4.27	4.96	5.84	5.30
MB16-43	43	468.39	16.11	15.83	15.46	16.63	16.50
PArel	20	4.63	2.00	2.85	2.32	2.09	2.16
RSE43	43	7.60	0.86	0.95	1.03	1.06	1.00
BH76	76	18.61	3.31	5.57	3.62	3.55	3.51
BHDIV10	10	45.33	4.38	4.39	4.58	4.58	4.38
BHPERI	26	20.87	1.69	1.62	1.84	1.64	1.71
BHROT27	27	6.27	0.62	0.58	0.63	0.73	0.73
INV24	24	31.85	3.49	2.29	3.57	3.45	5.04
PX13	13	33.36	2.70	2.81	3.10	2.62	2.17
WCPT18	18	34.99	2.90	3.00	3.58	3.23	3.02
ADIM6	6	3.36	0.25	0.06	0.12	0.11	0.09
AHB21	21	22.49	2.26	2.34	2.39	2.42	1.67
CARBHB12	12	6.04	1.26	1.66	1.32	1.21	1.30
CHB6	6	26.79	0.63	1.20	0.52	0.58	0.53
HAL59	59	4.59	1.35	1.36	1.54	1.36	1.31
HEAVY28	28	1.24	0.24	0.28	0.26	0.27	0.22
IL16	16	109.05	0.95	1.30	0.82	1.03	0.38
PNICO23	23	4.27	0.97	0.93	1.02	1.00	1.12
RG18	18	0.58	0.30	0.14	0.32	0.32	0.21
S22	22	7.30	0.50	0.45	0.59	0.52	0.44
S66	66	5.47	0.31	0.27	0.50	0.46	0.41
WATER27	27	81.17	4.86	4.84	10.60	8.48	6.01
ACONF	15	1.83	0.13	0.09	0.08	0.09	0.12
Amino20x4	80	2.44	0.29	0.26	0.28	0.25	0.24
BUT14DIOL	64	2.80	0.14	0.19	0.21	0.16	0.13
ICONF	17	3.27	0.49	0.51	0.46	0.37	0.44
IDISP	6	14.22	3.83	3.05	3.45	4.27	3.36
MCONF	51	4.97	0.39	0.35	0.41	0.49	0.51
PCONF21	18	1.62	0.61	0.69	0.54	0.53	0.55
SCONF	17	4.60	0.18	0.41	1.05	0.67	0.46
UPU23	23	5.72	0.52	0.55	0.44	0.46	0.47
NCIBLIND10	80	2.54	0.50	0.38	0.31	0.26	0.24
IONPI19	19	20.87	1.03	1.35	1.05	1.00	1.45
S30L	30	37.51	2.11	2.02	1.87	2.01	2.46
L7	7	16.21	1.45	1.43	1.29	1.23	1.64
CHAL336	336	14.09	2.51	2.76	2.60	2.56	2.12
X40x10	400	2.71	0.40	0.48	0.61	0.54	0.52
HB300SPX	300	4.58	0.69	0.72	0.85	0.78	0.75
R160x6	960	2.04	0.33	0.36	0.41	0.37	0.35
37CONF8	259	5.52	0.57	0.61	0.68	0.65	0.67
ACONF12	12	4.28	0.11	0.14	0.10	0.10	0.11
TMCONF5	16	3.15	0.43	0.35	0.33	0.34	0.29
MPCONF196	183	8.19	0.64	0.70	1.14	1.04	0.98
MOR41	41	31.18	4.62	5.06	4.29	4.35	4.41
ROST61	61	42.78	4.42	4.07	4.42	4.99	4.64
WCCR10	10	48.71	4.05	3.61	3.63	4.00	4.07
MOBH35	70	21.39	4.14	4.04	4.53	4.24	4.01
revMOBH35	58	20.48	0.63	0.74	0.76	0.68	0.72
MLA	24	48.82	2.58	2.78	3.18	3.06	2.80
TMBH	34	14.01	6.61	6.31	7.01	6.77	6.73

Table S13: Number of data points N, average of the absolute energies $\overline{|E|}$ and root-mean-square deviations (RMSD) in kcal mol[1] for each test set computed with r^2SCAN in combination with different basis sets. The respective D4 London dispersion correction was applied in all calculations.

			RMSD				
Set	N	$\overline{\|E\|}$	3c(STO)	3c(GTO)	TZP	TZ2P	QZ4P
AL2X6	6	35.88	1.25	1.17	1.45	1.75	1.66
ALK8	8	62.60	3.78	4.62	3.86	3.90	3.98
ALKBDE10	10	100.69	6.20	9.55	6.34	7.28	7.45
BH76RC	30	21.39	4.10	7.65	3.90	4.08	4.15
DC13	13	54.98	11.75	12.28	11.06	11.69	11.03
DIPCS10	10	654.26	5.65	5.97	5.92	5.69	5.83
FH51	51	31.01	3.50	4.71	3.69	3.41	3.30
G21EA	25	33.62	6.86	10.33	6.35	6.34	4.74
G21IP	36	257.61	5.48	6.90	5.48	5.46	5.68
G2RC	25	51.26	6.80	8.25	6.97	6.72	6.46
HEAVYSB11	11	58.02	4.72	5.21	4.62	4.13	4.38
NBPRC	12	27.71	2.44	2.25	3.88	1.90	2.06
PA26	26	189.05	2.61	2.67	2.72	2.52	3.24
RC21	21	35.70	6.69	7.52	7.62	6.87	7.08
SIE4x4	16	33.73	19.67	20.63	20.10	20.32	20.52
TAUT15	15	3.05	1.46	1.72	1.90	2.06	2.16
W4-11	140	306.91	5.81	7.88	6.37	4.98	5.15
YBDE18	18	49.28	5.73	7.79	5.57	4.74	3.94
BSR36	36	16.20	1.58	0.39	0.55	0.42	0.33
C60ISO	9	98.25	8.02	6.97	7.93	7.03	7.61
CDIE20	20	4.06	1.35	1.20	1.57	1.74	1.76
DARC	14	32.47	2.15	2.09	2.00	2.63	2.35
ISO34	34	14.57	1.97	1.92	1.96	2.11	1.96
ISOL24	24	21.92	4.93	4.22	4.90	5.81	5.26
MB16-43	43	468.39	16.07	15.78	15.39	17.45	18.96
PArel	20	4.63	1.98	2.78	2.28	2.11	2.20
RSE43	43	7.60	1.43	1.45	1.52	1.68	1.77
BH76	76	18.61	7.55	8.69	8.80	8.56	8.13
BHDIV10	10	45.33	6.00	5.92	6.87	6.87	6.60
BHPERI	26	20.87	5.28	4.97	5.67	5.13	4.98
BHROT27	27	6.27	0.80	0.82	0.81	0.98	1.02
INV24	24	31.85	3.44	2.39	3.53	3.38	5.23
PX13	13	33.36	7.23	7.05	11.24	10.01	9.21
WCPT18	18	34.99	5.21	5.28	7.90	7.17	6.84
ADIM6	6	3.36	0.53	0.16	0.23	0.19	0.20
AHB21	21	22.49	3.15	3.37	4.27	4.16	2.79
CARBHB12	12	6.04	1.81	2.24	1.98	1.82	1.67
CHB6	6	26.79	0.88	1.47	1.51	1.40	0.49
HAL59	59	4.59	1.59	1.65	1.83	1.64	1.50
HEAVY28	28	1.24	0.27	0.32	0.34	0.29	0.31
IL16	16	109.05	0.92	2.04	2.34	2.35	1.19
PNICO23	23	4.27	1.22	1.14	1.30	1.30	1.32
RG18	18	0.58	0.29	0.14	0.36	0.37	0.22
S22	22	7.30	0.50	0.44	0.78	0.66	0.56
S66	66	5.47	0.31	0.27	0.67	0.59	0.48
WATER27	27	81.17	6.04	6.59	16.14	13.18	9.10
ACONF	15	1.83	0.29	0.09	0.18	0.21	0.28

Amino20x4	80	2.44	0.29	0.26	0.29	0.25	0.24
BUT14DIOL	64	2.80	0.23	0.21	0.60	0.40	0.28
ICONF	17	3.27	0.48	0.50	0.53	0.41	0.49
IDISP	6	14.22	4.07	3.27	3.47	4.26	3.42
MCONF	51	4.97	0.54	0.59	0.68	0.65	0.63
PCONF21	18	1.62	0.59	0.71	0.61	0.54	0.58
SCONF	17	4.60	0.18	0.42	1.21	0.79	0.56
UPU23	23	5.72	0.51	0.54	0.44	0.48	0.58
NCIBLIND10	80	2.54	0.50	0.37	0.34	0.28	0.26
IONPI19	19	20.87	1.01	1.51	1.03	0.98	1.42
S30L	30	37.51	2.15	2.24	3.94	3.75	4.36
L7	7	16.21	1.43	1.33	1.32	1.30	1.96
CHAL336	336	14.09	2.86	3.32	3.12	3.31	2.63
X40x10	400	2.71	0.41	0.49	0.68	0.60	0.54
HB300SPX	300	4.58	0.79	0.86	1.21	1.11	0.95
R160x6	960	2.04	0.34	0.38	0.46	0.42	0.35
37CONF8	259	5.52	0.57	0.63	0.70	0.66	0.67
ACONF12	12	4.28	0.36	0.16	0.10	0.12	0.14
TMCONF5	16	3.15	0.44	0.36	0.33	0.33	0.29
MPCONF196	183	8.19	0.64	0.70	1.37	1.19	1.09
MOR41	41	31.18	4.73	5.01	4.64	4.52	4.39
ROST61	61	42.78	4.45	4.04	4.40	5.01	4.69
WCCR10	10	48.71	3.90	3.62	4.14	4.50	4.51
MOBH35	70	21.39	4.89	4.77	5.55	5.23	4.97
revMOBH35	58	20.48	0.70	0.82	0.76	0.69	0.76
MLA	24	48.82	3.12	3.26	3.88	3.68	3.45
TMBH	34	14.01	6.72	6.29	7.06	6.78	6.72

S3 Timings

Table S14: Wall-times and MADs in a single-point energy calculation for 8 datapoints from the S30L, MOR41, L7 and ROST61 benchmark sets. All calculations were carried out with ADF and include the D4 correction. Note that the wall-time for the semi-classical D4 and gCP corrections is negligible with 0.35 s and 0.15 s respectively. Computations were done on four Intel® Xeon® CPU E3-1270 v5@3.60GHz cores.

	Time / s	MAD / kcal mol^{-1}
r^2SCAN-3c	38691	2.99
r^2SCAN/TZP	39839	3.54
r^2SCAN/TZ2P	76212	3.92
PBE/TZ2P	31252	3.35
Libxc-PBE/TZ2P	69007	3.37
TPSS/TZ2P	71553	4.42
Libxc-TPSS/TZ2P	70270	4.42
PBE0/TZ2P	97261	2.57
B3LYP/TZ2P	97383	4.91
PBE/QZ4P	105040	3.72
Libxc-PBE/QZ4P	245774	3.74
r^2SCAN/QZ4P	242979	4.02
TPSS/QZ4P	255445	4.82
Libxc-TPSS/QZ4P	257273	4.82
PBE0/QZ4P	412206	2.80
Libxc-PBE0/QZ4P	504666	2.81
B3LYP/QZ4P	407446	4.86
SCAN/QZ4P	615106	3.67

Table S15: Wall-times of single-point energy calculations on different sizes of water clusters with r^2SCAN-3c and r^2SCAN in combination with different basis sets. Calculations were done on four Intel® Xeon® CPU E3-1270 v5@3.60GHz cores.

water clusters timing / s			
#	3c (STO)	TZP	TZ2P
25	1332	1314	2694
50	6444	6033	12107
75	16632	16981	32210
100	31543	34545	62569

Table S16: Wall-times of single-point energy calculations on different sizes of ammoniaclusters with r^{2}SCAN-3c and r^{2}SCAN in combination with different basis sets. Calculations were done on four Intel® Xeon® CPU E3-1270 v5@3.60GHz cores.

ammonia clusters timing / s			
#	3c (STO)	TZP	TZ2P
25	1827	1807	3744
50	10875	12904	25419
75	27652	30119	57307
100	60758	67108	126512

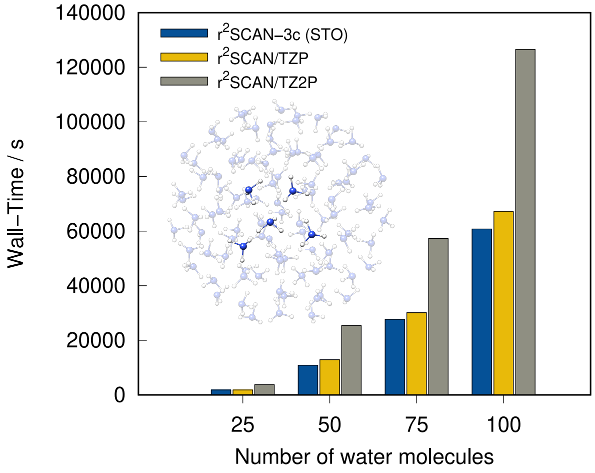

Figure S1: Timings for different sizes of ammonia clusters.
